# Selective Electrochemical Defluorinative Hydroxymethylation toward Difluoro-Substituted Alcohol Building Blocks

**DOI:** 10.1021/acs.orglett.6c01339

**Published:** 2026-06-02

**Authors:** Andrey Shatskiy, Márk Holczer, Johannes Winter, Ziwei Fan, Kevin Breitwieser, Helena Lundberg

**Affiliations:** § Department of Chemistry, 426712 KTH Royal Institute of Technology, SE-100 44 Stockholm, Sweden; ⊥ Department of Chemistry, Faculty of Science, University of Helsinki, FI-00560 Helsinki, Finland

## Abstract

Difluoromethylated compounds are of high importance for pharmaceutical and agrochemical industries; however, access to these structures remains limited due to the scarcity of reliable synthetic methods for their formation. This work presents a straightforward electrochemical synthesis of difluoromethyl-substituted alcohol building blocks via the selective monodefluorination of trifluoromethyl arenes with concomitant hydroxymethylation. The transformation proceeds with high chemoselectivity for a range of trifluoromethyl arene substrates under mild conditions. Mechanistic studies suggest that the reaction relies on reductive radical–polar crossover to furnish a key carbanion intermediate.

## Graphical abstract

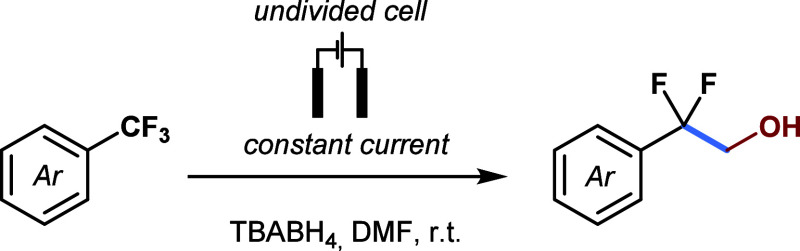

Fluorine-containing molecules are of great importance in the pharmaceutical and agrochemical sectors, with around 15–20% of all active compounds containing at least one fluorine atom.^1^ The prevalence of organofluorine compounds stems from the beneficial properties of the C–F bond with respect to physicochemical behavior, such as lipophilicity, hydrogen bonding and reactivity, as well as biological activity and metabolic stability. While trifluoromethyl groups have long been common motifs,^2^ their difluoro-substituted analogues have gradually become more prevalent, particularly as bioisosteres with modulated lipophilicity for hydroxy, thiol and methyl groups.^3^ Recently, this functionality has been targeted by a range of synthetic strategies proceeding via radical (open-shell) intermediates, including photoredox catalysis and electrosynthesis.^4^

Dating back to the 1950s, radical-mediated defluorination methods mostly relied on reductive monodefluorination of a trifluoromethyl group.^5^ An inherent challenge of this approach stems from the decrease of the bond dissociation energy (BDE) of the C–F bond with decreasing number of fluorides at the carbon center (Figure 1A).^6^ Meanwhile, mesolytic C–F bond cleavage in the key anion-radical intermediate is facilitated as the fluorination degree is reduced, leading to more facile cleavage of C–F bonds in compounds that are already partially defluorinated. In line with this trend, electrosynthetic protocols for complete defluorination of trifluoromethyl-decorated compounds to their methyl-substituted analogues are well established.^7^ At the same time, the reduction potential of the compound may also decrease with the number of fluorides at the carbon center,^8^ which can enable selective monodefluorination of trifluoromethyl arenes and ketones to their difluorinated analogues, as recently demonstrated by Lennox, Cheng, Wang and Xia (Figure 1B).^9^ However, the ability to intercept the reactive intermediates to furnish C-functionalized difluorinated products remains highly limited in electrochemical settings. Lyu and co-workers recently disclosed a protocol for monodefluorinative borylation of trifluoromethyl arenes with subsequent Matteson-type homologation.^10^ Clavel and Bordeau and co-workers demonstrated monodefluorinative silylation of the same class of starting materials,^11^ while similar silylation from trifluoromethylated carbonyl compounds was achieved by the Uneyama and Bordeau groups.^12^ In a brief communication, Troupel and co-workers disclosed a limited number of examples of C–C bond formation to furnish difluorinated tertiary alcohols, acetals and carboxylic acids from the corresponding trifluoromethyl arenes upon coupling with acetone, DMF and CO_2_.^13^ Additional contributions in this context were made by the groups of Guirado and Meanwell,^14^ while Senboku and co-workers demonstrated a closely related monodefluorination carboxylation strategy toward anti-inflammatory drugs.^15^ Finally, Cheng and co-workers disclosed a protocol for monodefluorinative alkylation of trifluoromethyl arenes with alkenes.^16^ In this work, the limited literature on monodefluorinative cross-couplings is expanded, showcasing that DMF can serve as a privileged electrophile to selectively furnish hydroxymethylated difluoromethyl arenes (Figure 1C)—a product class of high relevance for pharmaceutical purposes (Figure 1D).^17^

**Figure 1:**
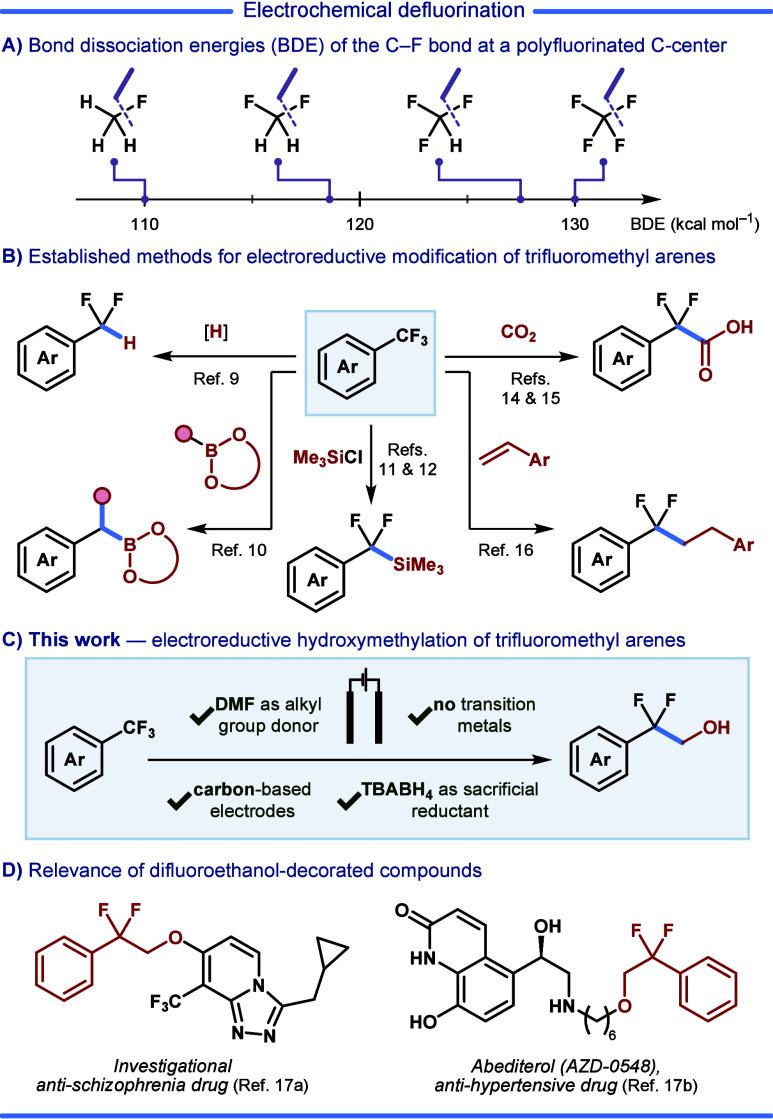
Electrochemical activation and functionalization of the C–F bond in trifluoromethyl-substituted organic compounds.

The optimization of reaction conditions commenced using (trifluoromethyl)­benzene (**1a**) as the model substrate (Table 1). Throughout optimization, 2,2-difluoro-2-phenylethan-1-ol (**2a**) was observed as the main product, and the corresponding monofluorinated derivative (**3a**) was observed as a minor side product. The remaining starting material could not be monitored due to its high volatility. The product of complete defluorination (2-phenylethanol) also was not observed. The reactions were monitored by quantitative ^1^H NMR analysis with 1,3,5-trimethoxybenzene as the internal standard (see the Supporting Information for details). The reaction was initially attempted on a 0.3 mmol scale in an undivided electrochemical cell with graphite electrodes under constant current conditions, using tetrabutyl­ammonium hexafluoro­phosphate (TBAPF_6_) and tetrabutyl­ammonium borohydride (TBABH_4_) as supporting electrolytes in anhydrous *N,N*-dimethylformamide (DMF) (Table 1, entries 1–2). The use of TBABH_4_ was based on its previously demonstrated successful dual role as a supporting electrolyte and a sacrificial reductant in net-reductive transformations.^18^ Similarly, DMF serves a dual role as a solvent and a donor of hydroxymethyl functionality in this reaction. To mitigate inherent risks for thermal runaway reactions associated with borohydride reagents in DMF,^19^ low concentrations and moderate loadings of the former were ensured throughout the synthetic work.

**Table 1:**
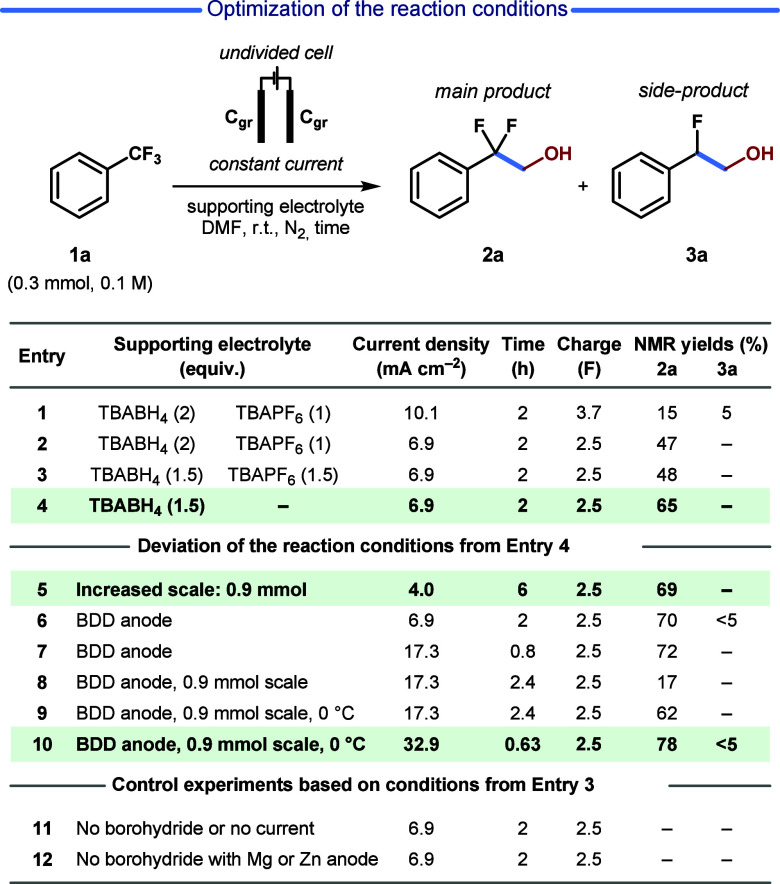
Optimization of the Reaction Conditions for the Model Reaction and Control Experiments^a^

a See the Supporting Information for details.

Conducting the reaction at a current density of 10.1 mA cm^–2^ resulted in formation of product **2a** in a modest yield of 15% (Table 1, entry 1), whereas a decrease in current density to 6.9 mA cm^–2^ increased the yield of **2a** to 47% (entry 2). Varying the ratio of TBAPF_6_ and TBABH_4_ while maintaining their combined concentration proved ineffective (entry 3), whereas a notable increase in the yield of **2a** to 65% was observed when TBAPF_6_ was removed (entry 4). Gratifyingly, the scale of the reaction could be increased up to 0.9 mmol with maintained yield of the desired product **2a** (69%, entry 5). Using boron-doped diamond (BDD) as anode material enabled formation of product **2a** in a similar yield (70%, entry 6), which was also maintained when the current density was increased up to 17.3 mA cm^–2^ (72%, entry 7). However, increasing the scale to 0.9 mmol severely hindered formation of **2a** (17%, entry 8). This problem was resolved by decreasing the reaction temperature to 0 °C, resulting in a yield of 62% (entry 9). Finally, adjustment of both the applied current density and the reaction time allowed formation of product **2a** in 78% yield (entry 10). Control experiments in the absence of either borohydride or electricity with either graphite, Zn or Mg anodes displayed no product formation (entries 11–12). Compared to graphite, BDD electrodes are less readily available and more costly, while higher current density and larger reaction scale provide notable practical benefits. Therefore, the two complementary sets of reaction conditions found in Table 1, entries 5 and 10, were used to probe the generality of the disclosed transformation.

The generality of the electroreductive defluorinative hydroxymethylation was investigated for a selection of trifluoromethyl-decorated aromatic substrates (Figure 2). The reactions were conducted on 0.9 mmol scale under optimized conditions featuring graphite cathode and anode at ambient temperature (Conditions A) or graphite cathode and BDD anode at 0 °C (Conditions B) and varying current density. Formation of product **2a** proceeded effectively under both conditions (65% and 78% yields under Conditions A and B). Curiously, formation of the para-methyl-decorated product **2b** was suppressed under Conditions A (18% yield), while the reaction proceeded effectively under Conditions B (50% yield). Electron-rich methoxy-substituted substrates demonstrated good compatibility with the disclosed transformation, delivering products **2c**–**2e** in 42–54% and 59–79% yields under Conditions A and B, respectively. Notably, even sterically encumbered ortho-substituted substrate **1e** effectively engaged in the reaction despite being potentially sensitive to intramolecular hydrogen-atom-transfer side reactions.^20^ A highly challenging methoxy-substituted pyridine-derived substrate **1f** was incompatible with Conditions A; however, the reaction was successfully realized under Conditions B to deliver the polyfunctionalized product **2f** in 44% yield. The substrate featuring a para-substituted phenolic ether delivered product **2g** with a notably higher yield under Conditions A (73%) relative to Conditions B (54%). Conversely, para-phenyl-substituted substrate furnished product **2h** in similar yields under both conditions (39% and 43% yields for Conditions A and B, respectively). The naphthalene-derived substrate **1i**, featuring an extended aromatic system, also proved compatible, delivering product **2i** in 27% and 41% yields under Conditions A and B, respectively. To our delight, moderately electron-poor fluoro-substituted substrates **1j** and **1k** effectively engaged in the reaction, despite being potentially sensitive to overreduction side reactions, delivering products **2j** and **2k** in 35% and 29% yields under Conditions A and in 65% and 59% yields under Conditions B. A considerably more electron-poor substrate **1l**, featuring a cyano group at the para-position, proved unsuccessful. Generally, the reaction displayed improved yields under Conditions B relative to Conditions A, which is attributed to both the decreased temperature and higher oxidative stability of the BDD anode, which ensures better selectivity of the oxidative counter reaction.

**Figure 2:**
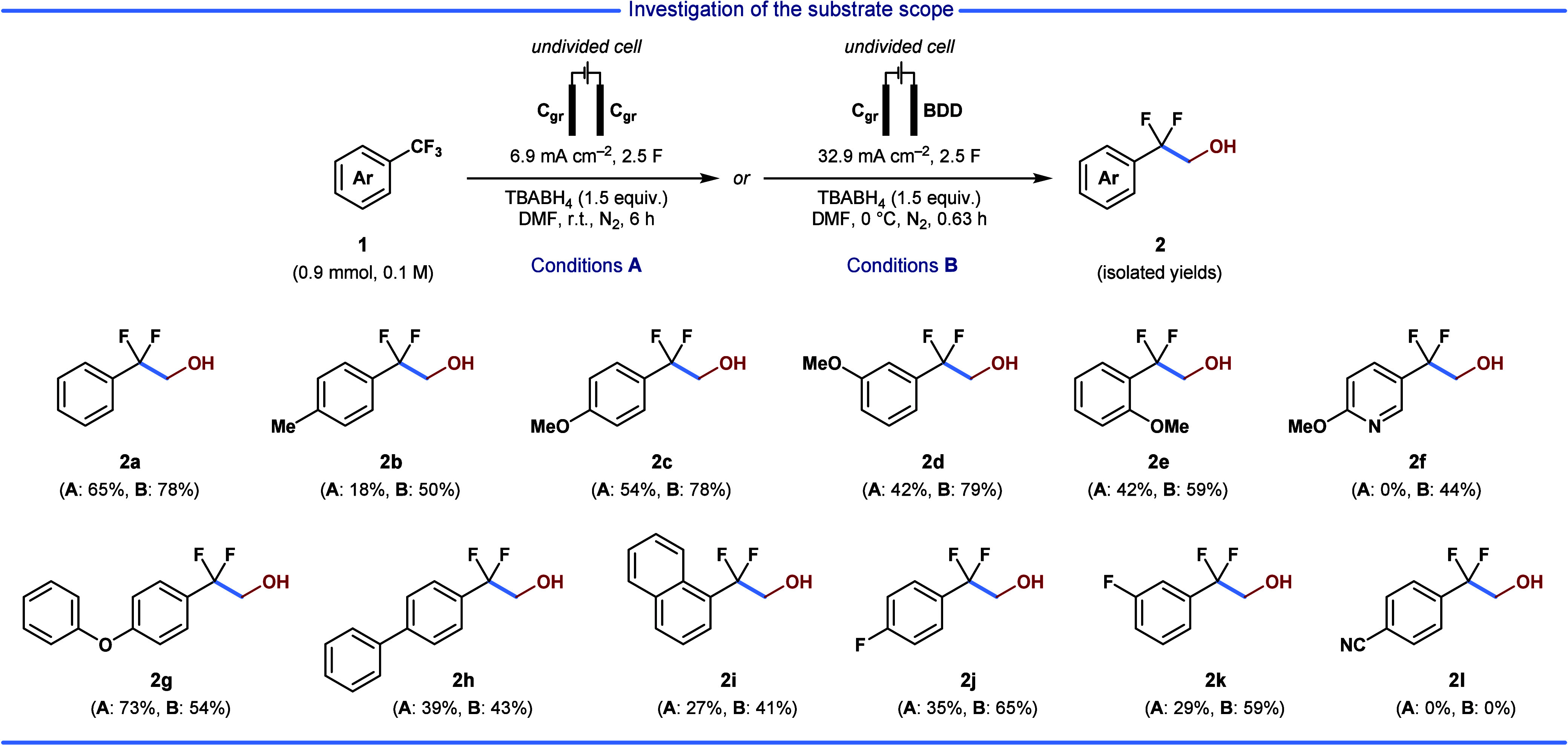
Investigation of the substrate scope of the electrochemical defluorinative hydroxymethylation reaction.

To probe the generality of the acceptor for the disclosed transformation, we explored the use of inert cosolvents and alternative carbonyl-containing compounds as coupling partners (Table S2). Under conditions similar to those of Table 1, entry 4, employing either toluene or THF as cosolvent allowed for selective formation of product **2a** in marginally lowered yields of ca. 45% (Table S2, entries 1–2). A range of alternative carbonyl acceptors was thereafter assessed either under neat conditions or with THF cosolvent (entries 3–9). Interestingly, C–C bond formation was hardly observed in these cases, with the exception of dimethyl carbonate (entry 4) and methyl formate (entry 8). The use of *N*-methyl-2-pyrrolidone (entry 5) and 1,3-dimethyl-2-imidazolidinone (entry 6) as acceptors promoted formation of mono- and difluoro-substituted side products **4a** and **5a**. These observations clearly highlight the selectivity of the disclosed electrosynthetic system, wherein DMF serves as a privileged carbonyl-containing acceptor. This finding is interesting in light of similar electroreductive protocols with trifluoromethyl arenes carried out in DMF that selectively furnish the hydrodefluorinated products.^7c,9c,9d^

Mechanistically, the transformation is proposed to be initiated via one-electron reduction at the cathode, converting the trifluoromethyl arene substrate into an unstable anion radical intermediate (Figure 3A). Elimination of a fluoride anion then delivers a difluoro-substituted benzylic radical, which undergoes a second one-electron reduction to deliver the corresponding carbanion.^8^ This species reacts with DMF through nucleophilic addition to the carbonyl functionality, followed by elimination of the dimethylamide anion, which leads to Hofmann elimination from a tetrabutyl­ammonium cation.^21^ Finally, the aldehyde intermediate is reduced to the primary alcohol product by borohydride. The outlined cathodic transformation is balanced by the oxidation of borohydride at the anode. Notably, the formation of an aldehyde intermediate was not observed in the control experiments in the absence of TBABH_4_ (Table 1, entries 11 and 12). Presumably, the aldehyde intermediate readily decomposes under these conditions, while in the presence of TBABH_4_ it is rapidly reduced to a more stable alcohol product. Additionally, the borane released from the anodic oxidation of TBABH_4_ is likely to further contribute to selectivity of the reaction through scavenging of the fluoride anions eliminated from the substrate.

**Figure 3:**
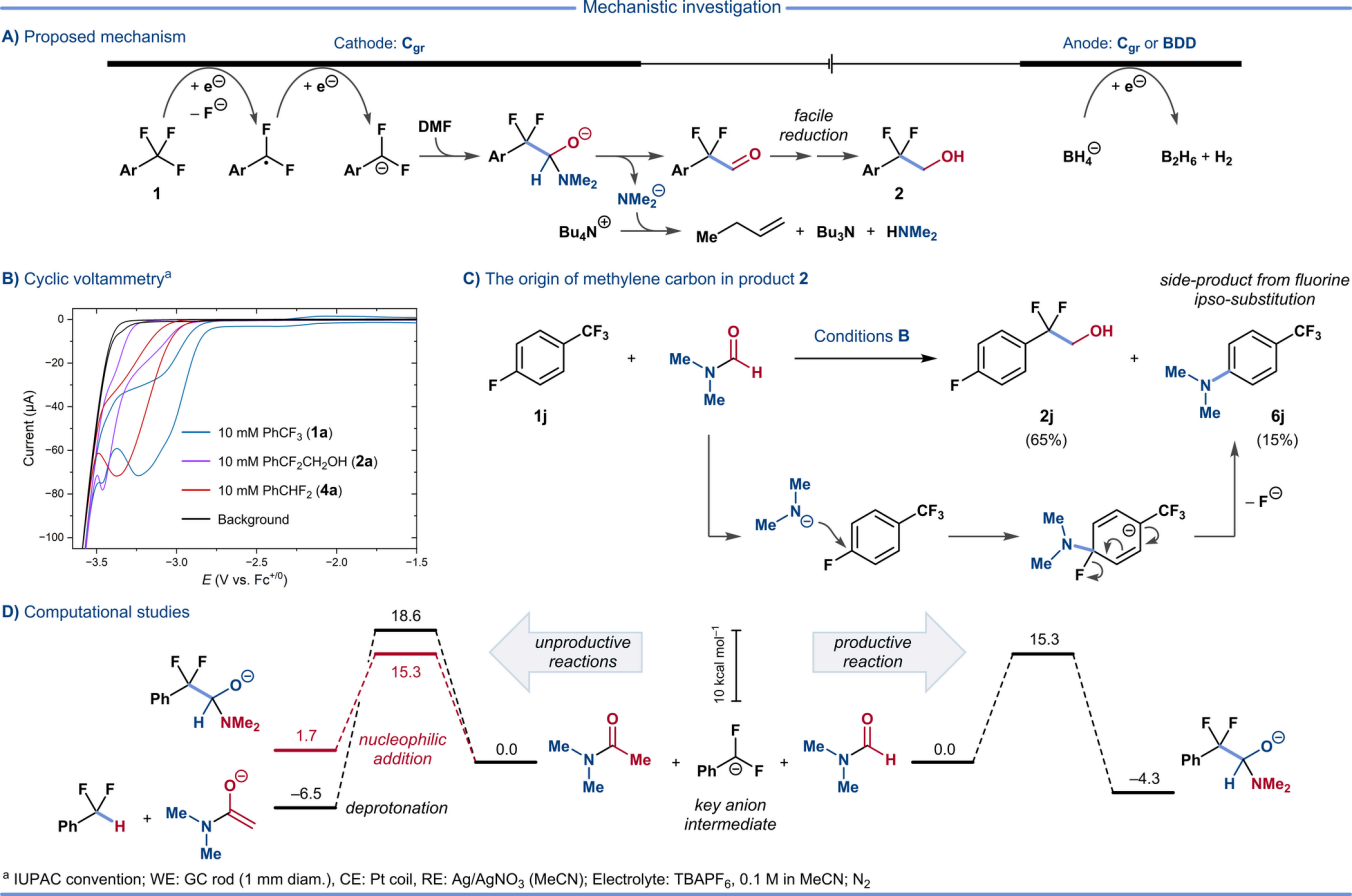
Proposed mechanism and mechanistic studies.

The use of DMF as an alkyl donor in C–C bond-forming reactions has been demonstrated under electrochemical conditions through two alternative mechanisms, where the new C–C bond is formed with the carbon center at either the *N*-methyl or the carbonyl functionality of DMF.^13,22^ The former mechanism relies on *in situ* oxidation of DMF to the iminium cation followed by nucleophilic addition. Such a reaction is less likely to proceed under the disclosed reductive conditions, rendering the proposed nucleophilic addition–elimination pathway more plausible. This is further supported by formation of side product **6j** during defluorinative hydroxymethylation of the para-fluorinated substrate **1j** (Figure 3C). Here, the eliminated dimethylamide anion, likely stabilized by the boron-based species present in solution, is trapped by the substrate through nucleophilic aromatic substitution, furnishing the corresponding *ipso*-substituted *N*,*N*-dimethylaniline derivative as the main side product.

The key C–C bond-forming step of the reaction was further investigated by density functional theory (DFT) calculations at the ZORA-ωB97X-D4­(SMD)/def2-TZVPP//r^2^SCAN-3c level of theory (Figure 3D) using Orca 6.1.0.^23,24^ A potential C–C bond-forming reaction between the difluoro-substituted benzylic radical and DMF appeared unfavorable both kinetically and thermodynamically (Δ*G* ^⧧^ = 28.6 kcal mol^–1^, Δ*G* = 25.9 kcal mol^–1^). Instead, the proposed nucleophilic addition of the difluoro-substituted benzylic anion to DMF demonstrated a considerably lower reaction barrier (Δ*G* ^⧧^ = 14.4 kcal mol^–1^) and was slightly exergonic (Δ*G* = −5.2 kcal mol^–1^), supporting the proposed mechanism. To gain further insight into the unique reactivity of DMF in this reaction relative to other carbonyl acceptors (Table S2), the nucleophilic addition step was also computed for the closest DMF analogue—*N*,*N*-dimethylacetamide (DMAc)—which failed to deliver the expected defluoroalkylated product under the benchmark conditions (Table S2, entry 7). While the reaction barrier for the nucleophilic addition to DMAc (Δ*G* ^⧧^ = 14.3 kcal mol^–1^) appeared to be of similar magnitude as for DMF, the overall reaction was found slightly endergonic (Δ*G* = 0.7 kcal mol^–1^). Furthermore, the α-carbonyl hydrogen in DMAc was found sufficiently acidic to be deprotonated by the difluoro-substituted benzylic carbanion with a reasonable barrier (Δ*G* ^⧧^ = 17.7 kcal mol^–1^) and considerable driving force (Δ*G* = −7.4 kcal mol^–1^). These findings rationalize why the expected defluoroalkylated product is not formed and elucidate the privileged nature of DMF as the coupling partner under the evaluated set of conditions.

To rationalize the high selectivity of the reaction toward formation of monodefluorinated products over further defluorinated analogues, cyclic voltammetry (CV) measurements were carried out. Assessment of the model substrate **1a** in DMF demonstrated an irreversible wave with *E*_pc_ = −3.2 V vs Fc^+^/Fc and a prominent shoulder at ca. −3.0 V vs Fc^+^/Fc, supporting the proposed two-electron reduction process (Figure 3B). The corresponding hydroxymethylated product **2a** displayed a considerably more negative reduction potential (*E*_pc_ = −3.5 V vs Fc^+^/Fc). Notably, the difference in the reduction potentials for **1a** and **2a** (Δ*E*_pc_ = 0.3 V) is significantly higher than for the substrate and product of the analogous hydrodefluorination reaction (Δ*E*_pc_ = 0.1 V) that produces (difluoromethyl)­benzene (**4a**) from **1a**. This difference in reduction potentials is likely responsible for the excellent chemoselectivity observed for the disclosed transformation that stands in contrast to that observed in many analogous hydrodefluorination protocols.^9^ Additionally, the onset of the reductive decomposition of DMF was observed at considerably lower potentials (Δ*E* > 0.5 V) relative to the onsets for reduction of **1a** and **2a**, suggesting that electroreductive solvent decomposition is unlikely to interfere with the desired transformation.

Herein, a straightforward electrochemical method is presented for the synthesis of difluoromethyl-substituted alcohol building blocks via the selective monodefluorination of trifluoromethyl arenes with concomitant hydroxymethylation. As such, the study adds to the limited synthetic literature on electrochemically triggered defluorinative cross-coupling, providing a route to difluoromethylated compounds that are relevant for pharmaceutical and agrochemical applications.

## Supplementary Material



## Data Availability

The data underlying this study are available in the published article and its Supporting Information.

## Notes

^1^H and ^13^C NMR spectra; experimental, analytical and computational details (PDF)

## †

Laboratory of Organic Electronics (LOE), Department of Science and Technology, Linköping University, SE-60174 Norrköping, Sweden
