# Supplementary material for: Selective Electrochemical Defluorinative Hydroxymethylation toward Difluoro-Substituted Alcohol Building Blocks
Source: Org Lett. 2026 Jun 2;28(23):7097–103. doi: 10.1021/acs.orglett.6c01339 (PMC13270633; doi:10.1021/acs.orglett.6c01339)
Supplement: Supplementary file 1 [file ol6c01339_si_001.pdf]

Supporting Information

Selective Electrochemical Defluorinative Hydroxymethylation towards Difluoro-Substituted Alcohol Building Blocks

Andrey Shatskiy,[a,b,‡] Márk A. Holczer,[a,‡], Johannes Winter,[a,‡] Ziwei Fan,[a] Kevin Breitwieser,[c] Helena Lundberg[a*]

[a] Department of Chemistry, KTH Royal Institute of Technology, SE-100 44 Stockholm, Sweden. e-mail: hellundb@kth.se

[b] Current affiliation: Laboratory of Organic Electronics (LOE), Department of Science and Technology, Linköping University, SE-60174 Norrköping, Sweden

[c] Department of Chemistry, Faculty of Science, University of Helsinki, FI-00560 Helsinki, Finland

‡ These authors contributed equally to this work

Contents

S1 General Information 3
S2 Electrochemical Equipment 3
S2.1 Electrochemical setup 4
S2.2 General Procedures 5
S3 Optimization 9
S4 Mechanistic Studies 13
S4.1 Electrochemical measurements 13
S4.2 Computational studies 13
S5 Experimental Procedures and Analytical Data 20
S6 Spectra 26
S7 References 48

S1 General Information

If not stated otherwise, all reactions were performed under ambient conditions and chemicals in analytical grade were used as purchased without further purification. THF or DMF was dried using a solvent dispensing system, where solvent is passed through activated alumina columns, stored under N_2 and over activated 3 Å molecular sieves when needed.

Chromatography

Thin layer chromatography (TLC) was performed using DC silica gel 60 F254 on aluminium plates (*Merck KGaA*, Darmstadt, Germany). Visualization of TLC plates was performed with UV lamp (λ = 254 nm). Flash column chromatography was conducted using 40-60 µm, 230-400 mesh, 60 Å silica (*SynLectro™ Sigma Aldrich/Merck KGaA*, Darmstadt, Germany) as stationary phase.

Nuclear Magnetic Resonance Spectroscopy (NMR)

NMR spectra were recorded using either a Bruker Avance II 400 MHz or a Bruker Avance 500 MHz spectrometer (*Bruker Corporation,* Billerica, USA) at 298 K (unless otherwise stated) using $CDCl_3$ as solvent. Chemical shifts are given in ppm relative to the residual solvent peak (^1H NMR: $CDCl_3$ δ 7.26; ^{13}C NMR: $CDCl_3$ δ 77.2) with multiplicity (br = broad, s = singlet, d = doublet, t = triplet, q = quartet, quin = quintuplet, sext = sextet, m = multiplet), coupling constants (in Hz) and integration.

High Resolution Mass Spectrometry (HR-MS)

High-resolution mass spectrometry analyses were performed using an Agilent 6530 quadrupole time of flight LC/MS with electrospray ionization (ESI) or a Thermo Scientific Q Exactive HF Hybrid Quadrupole-Orbitrap with atmospheric pressure chemical ionization (APCI).

S2 Electrochemical Equipment

Electrodes were either purchased from *IKA-Werke GmbH & Co. KG.* (8 · 52.5 · 2 mm, Staufen, Germany) or *SynLectro™ Sigma Aldrich/ Merck KGaA* (3 · 20 · 60 mm, Darmstadt, Germany). Prior to use, graphite electrodes were polished using sandpaper with different grain sizes (first 600 grid, followed by 1000 grid), rinsed with water and acetone and dried under vacuum overnight. Boron-doped diamond (BDD) electrodes were rinsed with acetone and dried with a paper towel prior to use. If the BDD electrodes were visibly coated with solid material they were cleaned by electrolyzing the electrodes with alternating polarity in aqueous 20% (*w*/*v*) sulfuric acid, applying 50 mA before electrolysis. Metal electrodes were polished using 600 grid sandpaper, rinsed with water and acetone and dried under vacuum overnight prior to use.

S2.1 Electrochemical setup

Electrochemical reactions were carried out using a multichannel galvanostat Aim TTi MX100QP (*Aim and Thurlby Thandar Instruments*, Huntingdon, United Kingdom). Commercial IKA® ElectraSyn vials and caps (Ident. No.: 0040003170) were used for electrochemical experiments. The electrochemical cell was sealed with PTFE tape and a rubber septum. The 100 mL electrolysis cell was constructed from a 100 mL laboratory bottle (*Avantor/VWR International AB*, Kista, Sweden), sealed with rubber septa and equipped with the corresponding electrode holders and electrodes *SynLectro™* (*Sigma Aldrich/ Merck KGaA*, Darmstadt, Germany).

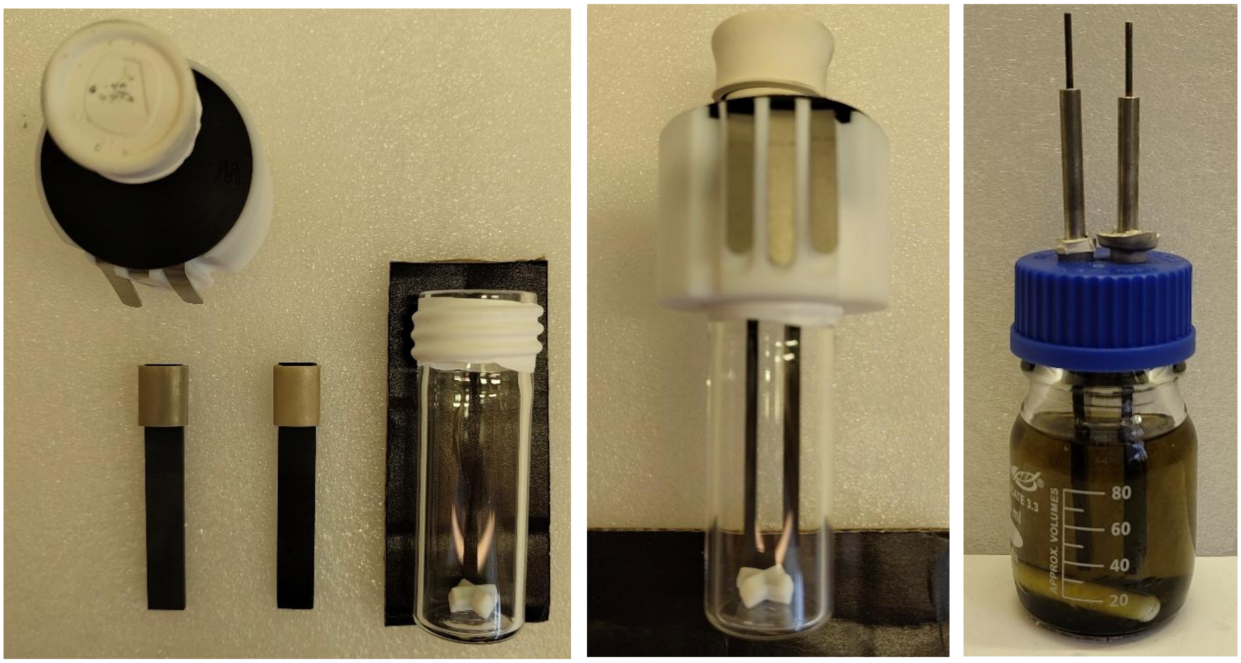

Figure S1: Electrochemical cells; left: disassembled cell, electrodes and lid; center: assembled cell; right: self-built 100 mL electrolysis cell.

S2.2 General Procedures

Caution: The starting materials and most of the products are volatile. The vacuum of the rotary evaporator after work-up and purification did not exceed <100 mbar.

GP1 Screening/Optimization of the Electrochemical Conditions; 3 mL Scale

An oven-dried (150 °C overnight) 5 mL IKA Electrasyn™ vial was charged with the corresponding supporting electrolyte. The vial was additionally sealed using PTFE tape to ensure air tightness and closed with the corresponding lid including the electrodes. The vial was evacuated and flushed with nitrogen three times before adding 2 mL of solvent. An oven-dried (150 °C overnight) 1.5 mL glass vial with a septum screw cap was purged with nitrogen and subsequently charged with the corresponding starting material (0.3 mmol, 1.0 equiv.). The starting material was dissolved in 0.5 mL of solvent, transferred to the electrochemical cell and the vial was rinsed once with 0.5 mL of solvent. The electrodes immersed 1.8-1.9 cm (resulting geometric area: 1.44-1.52 cm^2) into the solution. The current was adjusted according to the active electrochemical area. The electrolysis was performed under constant current conditions, and the current density was calculated by dividing the applied current by the immersed geometric area of the working electrode. After electrolysis, the contents of the cell were transferred into a 100 mL Erlenmeyer flask, the cell was rinsed with ethyl acetate or methyl *tert*-butyl ether and 5 mL of saturated ammonium chloride solution and 5 mL of water were added. The aqueous mixture was extracted three times using 10 mL of ethyl acetate or methyl *tert*-butyl ether. The combined organic layers were washed once with 10 mL of brine, dried over sodium sulphate or magnesium sulphate and the solvent was removed under reduced pressure.

The reactions were analyzed by qNMR by dissolving the crude mixtures after work-up in a stock solution of 1,3,5-trimethoxybenzene internal standard in $CDCl_3$ followed by ^1H NMR analysis. In a typical experiment, the crude residue from 0.3 mmol scale reaction is dissolved in 1.5 mL of $CDCl_3$ solution containing 16.82 mg of the internal standard, which corresponds to 1/3 equivalents of the internal standard relative to the starting material. The crude ^1H NMR spectrum is then analyzed by integrating the aromatic signals of the internal standards to 1.00 (δ 6.03 ppm, s, 3H), and the crude reaction yield for product **2a** is obtained by dividing the integral of the CH_2 functionality of the product (δ 3.91 ppm, t, *J* = 14.0 Hz, 2H) by two. The yield of the monofluorinated side-product **3a** is obtained directly from the integral of the benzylic CH signal (δ 5.52 ppm, ddd, *J* = 49.1, 7.7, 3.2 Hz, 1H).[1] Figure S2 shows a representative crude ^1H NMR spectrum for the reaction from entry 6, Table S1.

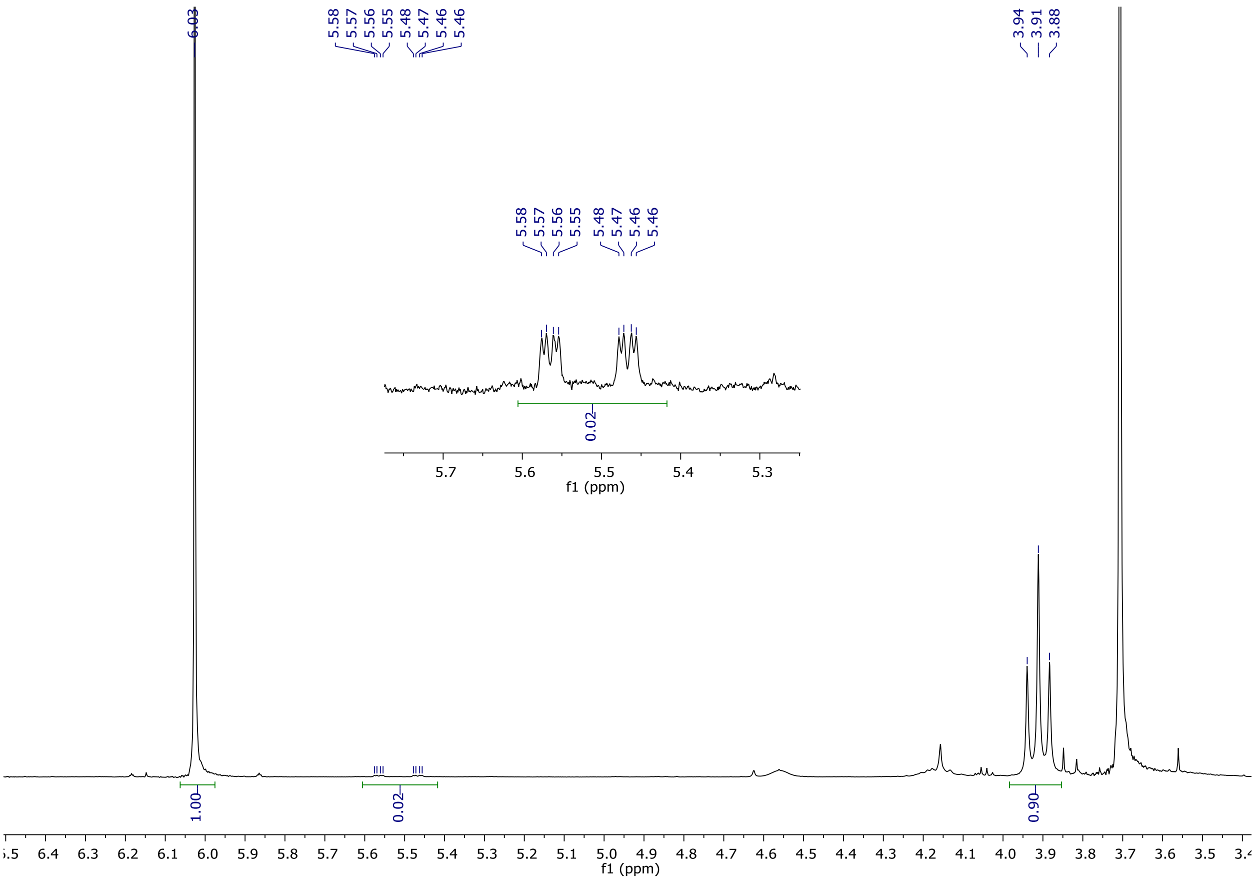

Figure S2: Representative ^{1}H NMR spectrum from analysis of the crude reaction mixture for entry 6, Table S1.

9 mL Scale

An oven-dried (150 °C overnight) 10 mL IKA Electrasyn™ vial was charged with the corresponding supporting electrolyte. The vial was additionally sealed using PTFE tape to ensure air tightness and closed with the corresponding lid including the electrodes. The vial was evacuated and flushed with nitrogen three times before adding 7 mL of solvent. An oven-dried (150 °C overnight) 1.5 mL glass vial with a septum screw cap was purged with nitrogen and subsequently charged with the corresponding starting material (0.9 mmol, 1.0 equiv.). The starting material was dissolved in 1 mL of solvent, transferred to the electrochemical cell and the vial was rinsed once with 1 mL of solvent. The electrodes immersed 3.0-3.3 cm (resulting geometric area: 2.40-2.64 cm^2) on a 9 mL scale into the solution. The current was adjusted according to the active electrochemical area. The electrolysis was performed under constant current conditions. After electrolysis, the contents of the cell were transferred into a 100 mL Erlenmeyer flask, the cell was rinsed with ethyl acetate or methyl *tert*-butyl ether and 5 mL of saturated ammonium chloride solution and 5 mL of water were added. The aqueous mixture was extracted three times using 10 mL of ethyl acetate or methyl *tert*-butyl ether. The combined organic layers were washed once with 10 mL of brine, dried over sodium sulphate or magnesium sulphate and the solvent was

removed under reduced pressure. 1,3,5-trimethoxybenzene was added to the crude mixture and the yield was determined by ^{1}H NMR analysis.

Caution: Mixing borohydride with DMF can result in a thermal runaway reaction. Reactions must be prepared with great caution.

GP2 Electrochemical Synthesis of Difluoroalcohols (9 mL Scale, Scope)

An oven-dried (150 °C overnight) 10 mL IKA Electrasyn™ vial was charged with *n*-tetrabutylammonium borohydride (347.4 mg, 1.35 mmol, 1.5 equiv.). The vial was additionally sealed using PTFE tape to ensure air tightness and closed with the lid including a boron-doped diamond (BDD)/graphite anode and a graphite cathode. The vial was evacuated and flushed with nitrogen three times before adding 7 mL of anhydrous DMF. An oven-dried (150 °C overnight) 1.5 mL glass vial with a septum screw cap was purged with nitrogen, charged with the corresponding starting material (0.9 mmol, 1.0 equiv.). The starting material was dissolved in 1 mL of anhydrous DMF, transferred into the electrochemical cell and the vial was rinsed once with 1 mL of anhydrous DMF. This resulted in a concentration of 0.1 M starting material. The electrodes immersed 3.0-3.3 cm (resulting geometric area: 2.40-2.64 cm^2) into the solution. The current was adjusted according to the active electrochemical area. When using BDD as anode material the reaction mixture was cooled to 0 °C prior to electrolysis. The electrolysis was performed under constant current conditions (current density j = 6.9 $mA \cdot cm^{-2}$ or 32.9 $mA \cdot cm^{-2}$) for 217.1 C (198–218 min for 6.9 $mA \cdot cm^{-2}$; 42–46 min for 32.9 $mA \cdot cm^{-2}$). After electrolysis, the contents of the cell were transferred into a 100 mL Erlenmeyer flask, the cell was rinsed with methyl *tert*-butyl ether and 5 mL of saturated ammonium chloride solution and 5 mL of water were added. The aqueous mixture was extracted three times using 10 mL of methyl *tert*-butyl ether. The combined organic layers were washed three times with 10 mL of 1 M aqueous hydrochloric acid, once with 10 mL of brine, dried over sodium sulphate and the solvent was removed under reduced pressure (≥ 100 mbar, bath temperature max. 40 °C). The products were purified by flash column chromatography using silica as stationary phase and different ratios of cyclohexane:ethyl acetate or diethyl ether:pentanes.

18 mL Scale

An oven-dried (150 °C overnight) 18 mL IKA Electrasyn™ vial was charged with *n*-tetrabutylammonium borohydride (693.8 mg, 2.7 mmol, 1.5 equiv.). The vial was additionally sealed using PTFE tape to ensure air tightness and closed with the lid including a boron-doped diamond (BDD) anode and a graphite cathode. The vial was evacuated and flushed with nitrogen three times before adding 15 mL of anhydrous DMF. An oven-dried (150 °C overnight) 1.5 mL glass vial with a septum screw cap was purged with nitrogen, charged with (trifluoromethyl)benzene (259.8 mg, 1.8 mmol, 1.0 equiv.). The starting material was dissolved in 2 mL of anhydrous DMF, transferred into the electrochemical cell and the vial was rinsed once with 1 mL of anhydrous DMF. This resulted in a concentration of 0.1 M starting material. The electrodes immersed 3.3 cm (resulting geometric area: 2.64 cm^2) into the solution. The current was adjusted according to the active electrochemical area. The reaction mixture was cooled to 0 °C prior to electrolysis. The electrolysis was performed under constant current conditions

(32.9 mA·cm^{-2}, 86.9 mA) for 434.2 C (92 min). Work-up and purification were performed as described above.

90 mL Scale

An oven-dried (150 °C overnight) 100 mL lab bottle was charged with *n*-tetrabutylammonium borohydride (3.482 g, 13.5 mmol, 1.5 equiv.). The bottle was additionally sealed using PTFE tape to ensure air tightness and closed with the lid including a boron-doped diamond (BDD) anode and a graphite cathode. The bottle was evacuated and flushed with nitrogen three times before adding 20 mL of anhydrous DMF and charged with (trifluoromethyl)benzene (1.316 g, 9 mmol, 1.0 equiv.) using a suitable syringe. The syringe was washed with 10 mL of anhydrous DMF and 60 mL of anhydrous DMF was added to the lab bottle to result in a concentration of 0.1 M starting material. The resulting solution was stirred at 0 °C (ice/water bath) for 15 min under a slight nitrogen overpressure. The electrodes (2·6 cm) immersed 4.5 cm (resulting geometric area: 9 cm^2) into the solution. The current was adjusted according to the active electrochemical area. The electrolysis was performed under constant current conditions (32.9 mA·cm^{-2}, 296.1 mA) for 2170.9 C (122 min). Work-up and purification were performed as described above.

S3 Optimization

Table S1. Optimization of the reaction conditions for substrate **1a**.

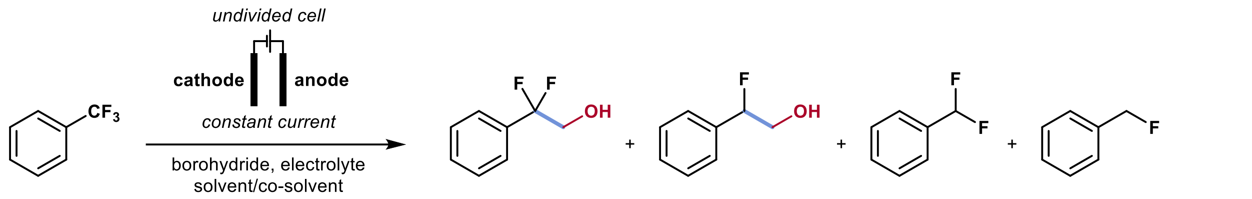

1a **2a** **3a** **4a** **5a**

Entry	Cathode	Anode	Borohydride (equiv.)	Electrolyte (equiv.)	Solvent (conc. (M))	Scale (mmol)	Temp.	Current density (mA cm^{-2})	Time (h) [a]	NMR yields (%)			
										2a	3a	4a	5a
					Initial observations								
**1**	C_{gr}	C_{gr}	$TBABH_4$ (2)	$TBAPF_6$ (1)	DMF (0.1)	0.3	r.t.	10.4	0.167	<5	–	–	–
									0.5	11	–	–	–
									1	35	–	–	–
									2	15	5	–	–
									3	–	<5	–	–
**2**	C_{gr}	C_{gr}	$TBABH_4$ (2)	$TBAPF_6$ (1)	DMF (0.1)	0.3	r.t.	6.9	1	17	–	–	–
									2	47	–	–	–
									3	36	<5	–	–
**3**	C_{gr}	C_{gr}	$TBABH_4$ (2)	$TBAPF_6$ (1)	DMF (0.1)	0.3	r.t.	3.5	2	32	–	–	–
									5	37	–	–	–
					Effect of borohydride and the supporting electrolyte								
**4**	C_{gr}	C_{gr}	–	$TBAPF_6$ (3)	DMF (0.1)	0.3	r.t.	6.9	2	–	–	–	–
**5**	C_{gr}	C_{gr}	$TBABH_4$ (0.5)	$TBAPF_6$ (2.5)	DMF (0.1)	0.3	r.t.	6.9	2	24	<5	–	–
**6**	C_{gr}	C_{gr}	$TBABH_4$ (1)	$TBAPF_6$ (2)	DMF (0.1)	0.3	r.t.	6.9	2	45	<5	–	–
**7**	C_{gr}	C_{gr}	$TBABH_4$ (1.5)	$TBAPF_6$ (1.5)	DMF (0.1)	0.3	r.t.	6.9	2	48	–	–	–
**8**	C_{gr}	C_{gr}	$TBABH_4$ (2)	$TBAPF_6$ (1)	DMF (0.1)	0.3	r.t.	6.9	2	47	–	–	–
**9**	C_{gr}	C_{gr}	$TBABH_4$ (2.5)	$TBAPF_6$ (0.5)	DMF (0.1)	0.3	r.t.	6.9	2	40	–	–	–
**10**	C_{gr}	C_{gr}	$TBABH_4$ (3)	–	DMF (0.1)	0.3	r.t.	6.9	2	42	–	–	–
**11**	C_{gr}	C_{gr}	$TBABH_4$ (1.5)	KPF_6 (1.5)	DMF (0.1)	0.3	r.t.	6.9	2	8	6	–	–
**12**	C_{gr}	C_{gr}	$NaBH_4$ (1.5)	KPF_6 (1.5)	DMF (0.1)	0.3	r.t.	6.9	2	8	<5	–	–
**13**	C_{gr}	C_{gr}	$LiBH_4$ (2)	–	DMF (0.1)	0.3	r.t.	6.9	2	9	<5	–	–
**14**	C_{gr}	C_{gr}	$TBABH_4$ (1)	–	DMF (0.1)	0.3	r.t.	6.9	2	52	<5	–	–

Entry	Cathode	Anode	Borohydride (equiv.)	Electrolyte (equiv.)	Solvent (conc. (M))	Scale (mmol)	Temp.	Current density (mA cm^{-2})	Time (h) [a]	NMR yields (%)			
										2a	3a	4a	5a
**15**	**C_{gr}**	**C_{gr}**	**$TBABH_4$ (1.5)**	**–**	**DMF (0.1)**	0.3	r.t.	**6.9**	**2**	**65**	**–**	**–**	**–**
**16**	C_{gr}	C_{gr}	$TBABH_4$ (2)	–	DMF (0.1)	0.3	r.t.	6.9	2	39	–	–	–
**17**	**C_{gr}**	**C_{gr}**	**$TBABH_4$ (1.5)**	**–**	**DMF (0.1)**	0.9	r.t.	**3.5**	**6**	**69**	**–**	**–**	**–**
					Concentration								
**18**	C_{gr}	C_{gr}	$TBABH_4$ (1.5)	–	DMF (0.05)	0.3	r.t.	6.9	1<br>2	38<br>20	<5<br>8	–<br>–	–<br>–
**19**	C_{gr}	C_{gr}	$TBABH_4$ (1.5)	–	DMF (0.2)	0.3	r.t.	6.9	2<br>3	26<br>36	–<br>–	–<br>–	–<br>–
					Cathode material [b]								
**20**	GC	C_{gr}	$TBABH_4$ (1.5)	–	DMF (0.1)	0.3	r.t.	6.9	2	55	–	–	–
**21**	St. S.	C_{gr}	$TBABH_4$ (1.5)	–	DMF (0.1)	0.3	r.t.	6.9	2	51	–	–	–
**22**	Ti	C_{gr}	$TBABH_4$ (1.5)	–	DMF (0.1)	0.3	r.t.	6.9	2	53	<5	–	–
					Anode material [b]								
**23**	C_{gr}	St. S.	$TBABH_4$ (1.5)	–	DMF (0.1)	0.3	r.t.	6.9	2	36	–	–	–
**24**	C_{gr}	BDD	$TBABH_4$ (1.5)	–	DMF (0.1)	0.3	r.t.	6.9	2	70	<5	–	–
**25**	C_{gr}	BDD	$TBABH_4$ (1.5)	–	DMF (0.1)	0.3	r.t.	10.4	1.3	71	–	–	–
**26**	C_{gr}	BDD	$TBABH_4$ (1.5)	–	DMF (0.1)	0.3	r.t.	13.8	1	70	–	–	–
**27**	C_{gr}	BDD	$TBABH_4$ (1.5)	–	DMF (0.1)	0.3	r.t.	17.3	0.8	72	–	–	–
**28**	C_{gr}	BDD	$TBABH_4$ (1.5)	–	DMF (0.1)	0.3	r.t.	20.7	0.67	62	<5	–	–
**29**	C_{gr}	BDD	$TBABH_4$ (1.5)	–	DMF (0.1)	0.3	r.t.	27.6	0.5	59	<5	–	–
**30**	C_{gr}	BDD	$TBABH_4$ (1.5)	–	DMF (0.1)	0.9	r.t.	8.7	2.4	17	–	–	–
**31**	C_{gr}	BDD	$TBABH_4$ (1.5)	–	DMF (0.1)	0.9	0 °C	8.7	2.4	62	–	–	–
**32**	C_{gr}	BDD	$TBABH_4$ (1.5)	–	DMF (0.1)	0.9	0 °C	16.4	1.28	69	–	–	–

Entry	Cathode	Anode	Borohydride (equiv.)	Electrolyte (equiv.)	Solvent (conc. (M))	Scale (mmol)	Temp.	Current density (mA cm^{-2})	Time (h) [a]	NMR yields (%)			
										2a	3a	4a	5a
**33**	C_{gr}	BDD	$TBABH_4$ (1.5)	–	DMF (0.1)	0.9	0 °C	26.3	47.6	73	–	–	–
**34**	C_{gr}	BDD	$TBABH_4$ (1.5)	–	DMF (0.1)	0.9	0 °C	32.9	38	78	<5	–	–
**35**	C_{gr}	BDD	$TBABH_4$ (1.5)	–	DMF (0.1)	0.9	0 °C	36.0	34	72	–	–	–
**36**	C_{gr}	BDD	$TBABH_4$ (1.5)	–	DMF (0.1)	0.9	0 °C	39.5	31	67	<5	–	–
Control experiments and other variations from the optimized conditions A, entry 15													
**37**	C_{gr}	C_{gr}	–	$TBAPF_6$ (1.5)	DMF (0.1)	0.3	r.t.	6.9	2	–	–	–	–
**38**	C_{gr}	Mg	–	$TBAPF_6$ (1.5)	DMF (0.1)	0.3	r.t.	6.9	2	–	–	–	–
**39**	C_{gr}	Zn	–	$TBAPF_6$ (1.5)	DMF (0.1)	0.3	r.t.	6.9	2	–	–	–	–
**40**	C_{gr}	C_{gr}	$TBABH_4$ (1.5)	$TBAPF_6$ (1.5)	DMF (0.1)	0.3	r.t.	0	2	–	–	–	–
**41**	C_{gr}	C_{gr}	$TBABH_4$ (1.5)	–	DMF (0.1)	0.3	0 °C	6.9	2	32	<5	–	–
**42**	C_{gr}	C_{gr}	$TBABH_4$ (1.5)	–	DMF (0.1) with 5 equiv. water	0.3	r.t.	6.9	2	–	–	–	–
Using co-solvents and alternative carbonyl acceptors													
**43**	C_{gr}	C_{gr}	$TBABH_4$ (1.5)	–	CH_2Cl_2/DMF 2:1 (0.1)	0.3	r.t.	6.9	2	–	–	–	–
**44**	C_{gr}	C_{gr}	$TBABH_4$ (1.5)	–	^{t}BuCN/DMF 2:1 (0.1)	0.3	r.t.	6.9	2	23	–	–	–
**45**	C_{gr}	C_{gr}	$TBABH_4$ (1.5)	–	$PhCH_3$/DMF 2:1 (0.1)	0.3	r.t.	6.9	2	50	<5	–	–
**46**	C_{gr}	C_{gr}	$TBABH_4$ (1.5)	–	$PhCH_3$/DMF 4:1 (0.1)	0.3	r.t.	6.9	2	47	<5	–	–
**47**	C_{gr}	C_{gr}	$TBABH_4$ (1.5)	–	THF/DMF 2:1 (0.1)	0.3	r.t.	6.9	2	40	–	–	–
**48**	C_{gr}	C_{gr}	$TBABH_4$ (1.5)	–	THF/DMF 4:1 (0.1)	0.3	r.t.	6.9	2	50	<5	–	–
**49**	C_{gr}	C_{gr}	$TBABH_4$ (1.5)	–	THF/DMF 8:1 (0.1)	0.3	r.t.	6.9	2	45	<5	–	–
**50**	C_{gr}	C_{gr}	$TBABH_4$ (1.5)	–	THF/NMF 8:1 (0.1)	0.3	r.t.	6.9	2	–	–	7	n.d.

Entry	Cathode	Anode	Borohydride (equiv.)	Electrolyte (equiv.)	Solvent (conc. (M))	Scale (mmol)	Temp.	Current density (mA cm^{-2})	Time (h) [a]	NMR yields (%)			
										2a	3a	4a	5a
**51**	C_{gr}	C_{gr}	$TBABH_4$ (1.5)	–	THF/DMC 8:1 (0.1)	0.3	r.t.	6.9	2	7	10	<5	<5
**52**	C_{gr}	C_{gr}	$TBABH_4$ (1.5)	–	THF/NMP 8:1 (0.1)	0.3	r.t.	6.9	2	–	–	18	24
**53**	C_{gr}	C_{gr}	$TBABH_4$ (1.5)	–	THF/DMI 8:1 (0.1)	0.3	r.t.	6.9	2	–	–	18	20
**54**	C_{gr}	C_{gr}	$TBABH_4$ (1.5)	–	DMAc (0.1)	0.3	r.t.	6.9	2	–	–	–	–
**55**	C_{gr}	C_{gr}	$TBABH_4$ (1.5)	–	Methyl formate (0.1)	0.3	r.t.	6.9	2	20	–	–	–
**56**	C_{gr}	C_{gr}	$TBABH_4$ (1.5)	–	*n*-Butyl formate (0.1)	0.3	r.t.	6.9	2	–	–	–	–

[a] each time-point represent a separate reaction; [b] GC = glassy carbon, St. S. = stainless steel, BDD = boron-doped diamond; n.d. = the yield could not be determined due to overlapping NMR signals

S4 Mechanistic Studies

S4.1 Electrochemical measurements

Cyclic voltammetry (CV) measurements were performed on potentiostat (CH Instruments Inc. 650E) in a one-compartment electrochemical cell with glassy carbon (⌀ 1 mm) as the working electrode, Pt coil as the counter electrode, and $Ag/AgNO_3$ (MeCN) as the reference electrode. The CV measurements were carried out under N_2 atmosphere on 10 mM solutions of analytes in anhydrous MeCN with 0.1 M $TBAPF_6$ as the supporting electrolyte. The potential was converted to Fc^+/Fc by recording the CV measurements on analogous solutions containing ferrocene. The CV measurements were recorded by swiping the applied potential from −0.5 to −3.6 to −0.5 V vs. Fc^+/Fc at 100 mV s^{-1} scan rate.

S4.2 Computational studies

All computations were carried out with ORCA v6.1.0.[2] The geometric parameters of all compounds were optimized using the r²SCAN-3c composite method[3] and all ground states were verified as true minima by the absence of imaginary frequences. Single point computations were carried out using the ωB97X-D4[4] hybrid functional in combination with the ZORA-def2-TZVPP basis set.[5] Tighter than default scf ("tightscf") and optimization ("tightopt") criteria were used. Scalar relativistic effects were modeled within the "Zeroth Order Regular Approximation" (ZORA)[6] and the RIJCOSX approximation[7] in combination with the SARC/J auxiliary basis set[8] were used to accelerate the computations. For single point computations, solvent effects were modeled using the SMD implicit solvent model with dimethylformamide (DMF) or dimethylacetamide (DMA) as solvent.[9]

In the case of the radical reaction, the transition state was obtained using Orca's implementation of the nudged-elastic-band (NEB) method.[10]

Standard state correction:

As Gibbs free energies are computed as gases at 1 atm pressure, for the calculation of energy barriers etc. they are corrected to reaction conditions according to Henry's Law as described in literature before,[11] where T is the temperature and R the universal gas constant. The assumed concentration is the standard concentration of 1 M at all times except for DMF, for which a concentration of 12.92 M for neat DMF, derived from the molecular weight of 73.094 g mol^{-1} and a density of 0.948 g mL^{-1}, and for DMA (concentration of 10.76 M from 87.120 g mol^{-1} and 0.937 g mL^{-1}) is assumed:[12]

$$G_{corr} = G + RT \cdot \ln\left(c \cdot 24.47\ L \cdot mol^{-1}\right)$$

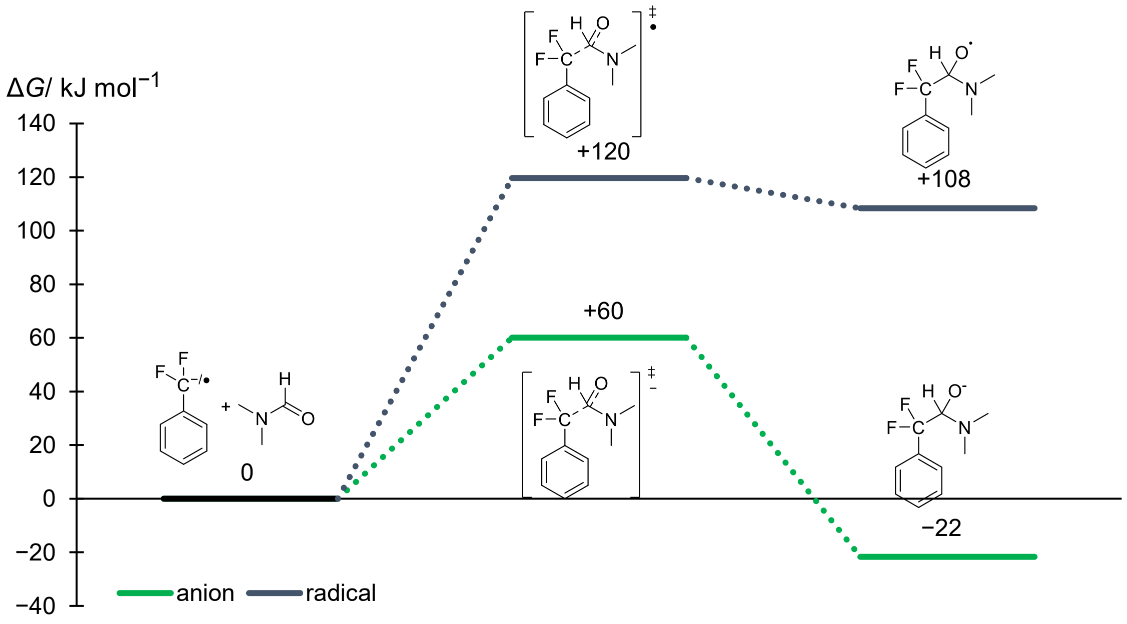

Figure S2: Reaction coordinate for the reaction of DMF with a difluorobenzyl anion (green) or difluorobenzyl radical (blue). ZORA-ωB97X-D4(SMD=DMF)/def2-TZVPP//r²SCAN-3c.

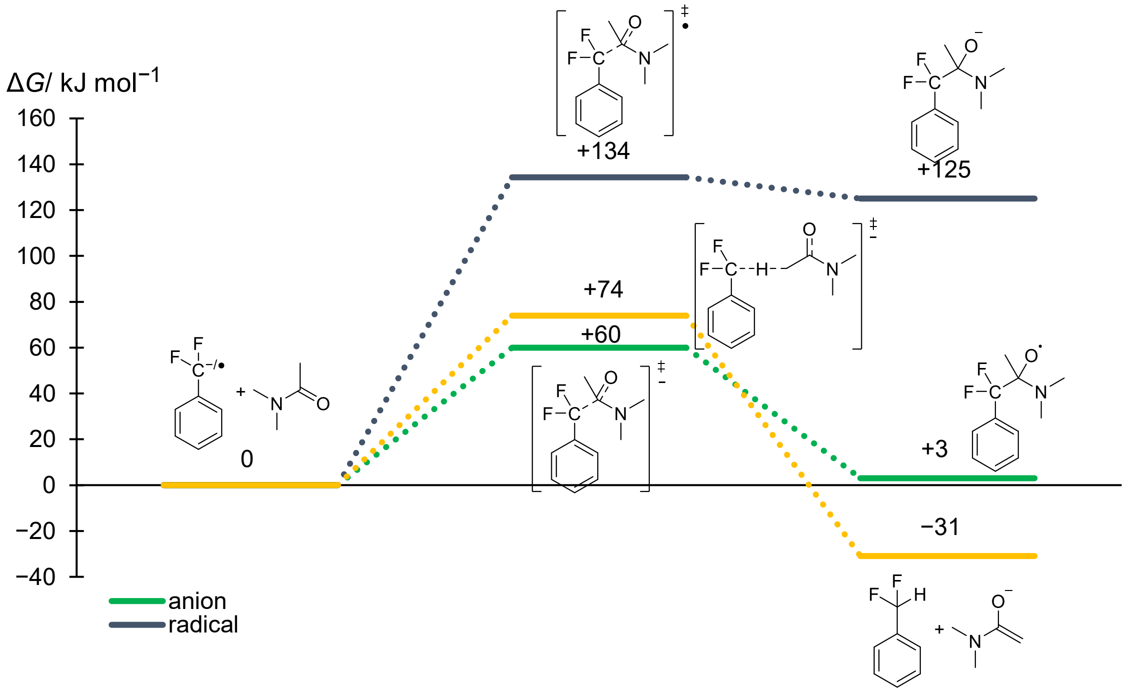

Figure S3: Reaction coordinate for the reaction of DMA with a difluorobenzyl anion (green: addition, orange: deprotonation) or difluorobenzyl radical (blue). ZORA-ωB97X-D4(SMD=DMA)/def2-TZVPP//r²SCAN-3c.

Table S2: Single point energies *E*, and Gibbs energies *G* for ground state molecules in gas phase (r²SCAN-3c) and using the SMD(DMF) implicit solvent model (ωB97X-D4).

	E(r²SCAN-3c)/ Eh	*G*(r²SCAN-3c)/ Eh	*E*(ωB97X-D4)/ Eh	*G*(ωB97X-D4)/ Eh	G_{corr}(ωB97X-D4)/ kJ mol^{-1}
DMF	−248.449361	−248.376968	−248.929254	−248.856861	−653359.85
$PhCF_2^-$	−469.359171	−469.294303	−470.301614	−470.236746	−1234599.47
$PhCF_2^•$	−469.331112	−469.265216	−470.185239	−470.119343	−1234291.22
$PhCF_2$–DMF^-	−717.845616	−717.682342	−719.259706	−719.096432	−1887981.00
$PhCF_2$–$DMF^•$	−717.764240	−717.601521	−719.092224	−718.929504	−1887542.73

Table S3: Imaginary frequencies, single point energies *E*, Gibbs energies *G* for transition states in gas phase (r²SCAN-3c) and using the SMD(DMF) implicit solvent model (ωB97X-D4).

	imaginary freq./ Hz	*E*(r²SCAN-3c)/ Eh	*G*(r²SCAN-3c)/ Eh	*E*(ωB97X-D4)/ Eh	*G*(ωB97X-D4)/ Eh	G_{corr}(ωB97X-D4)/ kJ mol^{-1}
TS($PhCF_2^-$+DMF)	59.96i	−717.825781	−717.665523	−719.225542	−719.065284	−1887899.22
TS($PhCF_2^•$+DMF)	67.19i	−717.760242	−717.598111	−719.087330	−718.925199	−1887531.43

Table S4: Single point energies *E*, and Gibbs energies *G* for ground state molecules in gas phase (r²SCAN-3c) and using the SMD(DMA) implicit solvent model (ωB97X-D4).

	E(r²SCAN-3c)/ Eh	*G*(r²SCAN-3c)/ Eh	*E*(ωB97X-D4)/ Eh	*G*(ωB97X-D4)/ Eh	G_{corr}(ωB97X-D4)/ kJ mol^{-1}
DMA	−287.755335	−287.657374	−288.313539	−288.215578	−756696.68
DMA-enolate	−287.138861	−287.054505	−287.774911	−287.690555	−755324.12
$PhCF_2H$	−469.978552545736	−469.89758363	−470.852394839123	−470.771426	−1236003.27
$PhCF_2^-$	−469.359171	−469.294303	−470.301733	−470.236865	−1234599.78
$PhCF_2^•$	−469.331112	−469.265216	−470.185323	−470.119427	−1234291.45
$PhCF_2$–DMA^-	−757.147039	−756.956596	−758.636454	−758.446011	−1991293.39
$PhCF_2$–$DMA^•$	−757.068445	−756.878328	−758.472237	−758.282119	−1990863.09

Table S5: Imaginary frequencies, single point energies *E*, Gibbs energies *G* for transition states in gas phase (r²SCAN-3c) and using the SMD(DMA) implicit solvent model (ωB97X-D4).

	imaginary freq./ Hz	*E*(r²SCAN-3c)/ Eh	*G*(r²SCAN-3c)/ Eh	*E*(ωB97X-D4)/ Eh	*G*(ωB97X-D4)/ Eh	G_{corr}(ωB97X-D4)/ kJ mol^{-1}
TS($PhCF_2^-$+DMA)	61.97i	−757.129621	−756.943962	−758.609999	−758.424340	−1991236.49
TS($PhCF_2^•$+DMA)	446.06i	−757.063468	−756.875249	−758.466808	−758.278589	−1990853.82
TS($PhCF_2^-$+DMA deprotonation)	1081.94i	−757.129380070224	−756.94839738	−758.599998515574	−758.419016	−1991222.52

xyz coordinates

DMF

C	-1.083859	3.600568	-1.516961
O	-0.205153	3.085533	-0.847032
N	-0.904860	4.598353	-2.425753
C	0.418063	5.146470	-2.672553
H	1.131729	4.625343	-2.031195
H	0.439593	6.220454	-2.444991
H	0.703488	5.007861	-3.723784
C	-2.011363	5.153706	-3.179514
H	-2.123427	6.228457	-2.980942
H	-2.940630	4.651290	-2.894939
H	-1.860224	5.017822	-4.259212
H	-2.148402	3.293208	-1.445264

DMA

C	-0.908984	3.427566	-1.817953
O	0.086842	2.871497	-1.367900
N	-0.843319	4.652198	-2.433160
C	0.455986	5.273970	-2.648433
H	1.210645	4.707821	-2.101656
H	0.441761	6.311265	-2.290807
H	0.714376	5.279873	-3.717208
C	-1.960183	5.308382	-3.090778
H	-1.936825	6.383117	-2.870515
H	-2.915354	4.917218	-2.741556

H	-1.912957	5.187915	-4.184385
C	-2.279537	2.783114	-1.696657
H	-2.738010	2.609043	-2.675949
H	-2.967012	3.397420	-1.105329
H	-2.141694	1.826277	-1.193975

DMA-enolate

C	-0.972182	3.440279	-1.710017
O	0.070720	2.992484	-1.155878
N	-0.894303	4.852581	-2.129835
C	0.404607	5.229370	-2.654679
H	1.168398	4.726639	-2.055780
H	0.542332	6.320770	-2.595490
H	0.545115	4.930600	-3.717760
C	-1.974880	5.395928	-2.919739
H	-1.818604	6.476482	-3.063115
H	-2.928362	5.258757	-2.398247
H	-2.069565	4.932675	-3.926043
C	-2.164657	2.762834	-1.926147
H	-2.236764	1.748272	-1.549901
H	-3.009405	3.164758	-2.469304

PhCF2H

C	2.104108	2.922106	-1.048457
C	0.771169	3.020355	-1.436147
C	2.532498	1.841728	-0.284662
C	1.624047	0.852293	0.088831
C	0.287328	0.951091	-0.295601
C	-0.135183	2.033694	-1.059100
H	2.815701	3.686736	-1.345516
H	0.439419	3.863681	-2.034761
H	3.573642	1.758508	0.010195
H	-0.415882	0.175447	-0.009420
H	-1.174974	2.103697	-1.364442
C	2.072601	-0.295151	0.951190
F	3.392946	-0.591149	0.714523
F	1.341736	-1.424242	0.670032
H	1.971368	-0.103866	2.029755

PhCF2−

C	2.102564	2.914021	-1.028586
C	0.757446	3.054454	-1.399853
C	2.547507	1.812640	-0.319585
C	1.654685	0.773016	0.075111
C	0.296650	0.916863	-0.335742
C	-0.127893	2.026438	-1.044457
H	2.821573	3.681701	-1.314809
H	0.415622	3.923394	-1.955152
H	3.595639	1.726389	-0.047408
H	-0.409006	0.132951	-0.075669
H	-1.174258	2.092084	-1.342887
C	2.065758	-0.275496	0.945892
F	3.421451	-0.640899	0.763455
F	1.338093	-1.473357	0.745222

PhCF2●

C	2.108217	2.922208	-1.024797
C	0.759414	3.049136	-1.372311
C	2.565782	1.803053	-0.357529
C	1.660093	0.761548	-0.017792
C	0.290856	0.897300	-0.374252
C	-0.137276	2.028025	-1.041648
H	2.809254	3.710662	-1.282622
H	0.411186	3.933492	-1.895810
H	3.612962	1.709155	-0.090779
H	-0.408495	0.107887	-0.120721
H	-1.184912	2.120129	-1.312889
C	2.099751	-0.354751	0.691906
F	3.380222	-0.606747	0.936728
F	1.338777	-1.416897	0.928048

PhCF2–DMF−

C	0.966290	1.756407	0.698874
C	0.403882	2.141331	-0.516679
C	1.254945	0.419136	0.952145
C	0.971495	-0.548889	-0.019799
C	0.396079	-0.165561	-1.230915
C	0.121754	1.175453	-1.481982
H	1.174722	2.504452	1.460420
H	0.182554	3.188476	-0.710252
H	1.658457	0.089890	1.902830
H	0.149390	-0.928204	-1.961813
H	-0.323466	1.467443	-2.430356
C	1.170812	-1.981306	0.335291
F	2.456761	-2.149875	0.844846
F	1.145561	-2.773231	-0.808319
C	0.168309	-2.536065	1.429124
N	-1.218033	-2.524181	0.740444
O	0.264757	-1.913232	2.567945
H	0.444186	-3.633191	1.430209
C	-2.002994	-1.378715	1.193539
H	-2.233992	-1.417340	2.273789
H	-2.944849	-1.344528	0.624958

H	-1.457523	-0.447843	1.016187
C	-1.943784	-3.748515	1.039064
H	-2.929356	-3.733843	0.548850
H	-2.113068	-3.900645	2.127406
H	-1.394973	-4.617595	0.656453

PhCF2–DMF●

C	1.182854	1.784718	0.474171
C	0.469958	2.057795	-0.690151
C	1.417304	0.469719	0.859671
C	0.937735	-0.576028	0.071016
C	0.218911	-0.306849	-1.092272
C	-0.010112	1.011465	-1.472367
H	1.558151	2.598988	1.086881
H	0.289266	3.086514	-0.988491
H	1.959948	0.246228	1.771749
H	-0.155443	-1.126412	-1.696596
H	-0.564063	1.220889	-2.382755
C	1.137883	-1.988563	0.532349
F	2.380092	-2.161878	1.066691
F	1.036617	-2.873932	-0.516922
C	0.090260	-2.418570	1.668875
N	-1.229316	-2.400771	1.034241
O	0.245664	-1.693098	2.738521
H	0.362727	-3.490507	1.845685
C	-2.156687	-1.416726	1.543242
H	-2.573849	-1.704482	2.523002
H	-2.977868	-1.293006	0.828927
H	-1.630762	-0.466872	1.687327
C	-1.792255	-3.643164	0.548559
H	-2.544009	-3.433767	-0.221207
H	-2.280574	-4.220290	1.353649
H	-1.004515	-4.261572	0.108463

PhCF2–DMA−

C	0.941347	1.802368	0.660616
C	0.319509	2.165233	-0.532652
C	1.253714	0.470928	0.917035
C	0.933112	-0.511799	-0.025960
C	0.304871	-0.150396	-1.216099
C	0.005238	1.184671	-1.471957
H	1.177026	2.562307	1.402090
H	0.077199	3.207330	-0.728532
H	1.702749	0.158292	1.852561
H	0.033133	-0.925926	-1.923853
H	-0.485780	1.459496	-2.402635
C	1.180813	-1.942797	0.323184
F	2.487608	-2.051890	0.799816
F	1.173806	-2.715800	-0.837327
C	0.200860	-2.530836	1.443102
N	-1.192419	-2.455569	0.730610
O	0.292612	-1.844510	2.549159
C	0.619837	-4.023524	1.611107
C	-1.937673	-1.290123	1.203241
H	-2.274707	-1.390275	2.251907
H	-2.819038	-1.149790	0.558141
H	-1.324251	-0.389349	1.154742
C	-2.040361	-3.626357	0.878744
H	-2.994975	-3.445966	0.360924
H	-2.289499	-3.871135	1.934666
H	-1.587892	-4.513241	0.423580
H	0.582740	-4.622242	0.692894
H	-0.022218	-4.473979	2.373599
H	1.644049	-4.022560	1.991206

PhCF2–DMA●

C	1.125150	1.813831	0.430414
C	0.390266	2.066415	-0.724514
C	1.389222	0.505610	0.820772
C	0.915653	-0.554772	0.048577
C	0.179328	-0.305216	-1.107946
C	-0.080278	1.005862	-1.493307
H	1.494703	2.638731	1.032392
H	0.186348	3.089614	-1.026962
H	1.949672	0.298619	1.725684
H	-0.186812	-1.134880	-1.703612
H	-0.650017	1.198659	-2.397673
C	1.148425	-1.963222	0.517227
F	2.410987	-2.098088	1.023338
F	1.062062	-2.847242	-0.539014
C	0.123330	-2.404449	1.662274
N	-1.207991	-2.336802	1.015999
O	0.260710	-1.607665	2.693198
C	0.465602	-3.865793	2.092404
C	-2.103669	-1.343731	1.562680
H	-2.521747	-1.645525	2.538274
H	-2.925208	-1.173558	0.859208
H	-1.547834	-0.415488	1.736840
C	-1.821349	-3.485520	0.383859
H	-2.634647	-3.141661	-0.265168
H	-2.253892	-4.192404	1.114219
H	-1.097587	-4.021054	-0.233440
H	0.474462	-4.544895	1.238237

H	-0.278350	-4.183744	2.825737
H	1.449849	-3.847342	2.558519

TS(PhCF2−+DMF)

C	1.438113	1.035037	1.391156
C	0.681778	1.886307	0.578556
C	1.657160	-0.287240	1.039033
C	1.125481	-0.823580	-0.160807
C	0.362196	0.051370	-0.977024
C	0.151322	1.369901	-0.608902
H	1.874069	1.416891	2.313264
H	0.509921	2.920708	0.862647
H	2.251010	-0.931489	1.680745
H	-0.060850	-0.336862	-1.899249
H	-0.435768	2.013950	-1.261667
C	1.170827	-2.225874	-0.457862
F	2.266344	-2.878258	0.146661
F	1.272816	-2.502122	-1.812811
C	-1.071109	-3.099422	-0.114277
N	-0.903711	-3.808454	1.096123
O	-1.338677	-3.665252	-1.176674
H	-1.325475	-2.038342	0.078961
C	-0.381760	-5.156704	0.957986
H	-0.576315	-5.726631	1.876377
H	0.705817	-5.151114	0.764898
H	-0.877836	-5.640049	0.112512
C	-0.322006	-3.060780	2.194723
H	0.778982	-3.048887	2.150195
H	-0.636370	-3.494086	3.155108
H	-0.665414	-2.020996	2.153532

TS(PhCF2●+DMF)

C	0.922829	3.464270	0.358759
C	0.385594	3.948302	-0.829636
C	1.013360	2.093489	0.576217
C	0.570035	1.206617	-0.404873
C	0.016307	1.689744	-1.591418
C	-0.065349	3.059234	-1.802950
H	1.277114	4.154154	1.118839
H	0.314723	5.019157	-0.997167
H	1.442312	1.714678	1.498019
H	-0.356592	0.988932	-2.329413
H	-0.492324	3.435457	-2.727709
C	0.637357	-0.265655	-0.173392
F	1.556055	-0.575421	0.793875
F	0.999485	-0.945054	-1.294168
C	-0.859321	-0.876109	0.291770
N	-0.632587	-2.261988	0.610392
O	-1.695084	-0.586948	-0.643078
H	-0.989741	-0.322958	1.257026
C	-0.961397	-3.267556	-0.367223
H	-1.871925	-3.821549	-0.085685
H	-0.138122	-3.988874	-0.463972
H	-1.155198	-2.777234	-1.324521
C	-0.086868	-2.685152	1.875303
H	0.870210	-3.211689	1.743993
H	-0.781323	-3.368332	2.389655
H	0.080449	-1.819515	2.521356

TS(PhCF2−+DMA)

C	1.315464	1.744448	0.667733
C	0.698507	2.050933	-0.547750
C	1.597565	0.431496	1.015196
C	1.270494	-0.638884	0.148886
C	0.639814	-0.315244	-1.075897
C	0.366654	1.000185	-1.410068
H	1.589062	2.546386	1.351997
H	0.475174	3.080532	-0.814277
H	2.079625	0.208321	1.962514
H	0.352098	-1.122232	-1.741528
H	-0.120929	1.215745	-2.359271
C	1.395959	-2.010291	0.565424
F	2.446296	-2.169914	1.508265
F	1.745590	-2.861496	-0.506839
C	-1.000218	-2.641587	1.237098
N	-0.807381	-4.038661	1.291207
O	-1.688924	-2.150720	0.335804
C	-0.795313	-1.878570	2.534113
C	-0.967595	-4.735392	0.020770
H	-1.171964	-5.796932	0.214525
H	-0.065042	-4.652370	-0.605734
H	-1.804746	-4.292041	-0.521128
C	0.263659	-4.591458	2.105444
H	1.257671	-4.384919	1.678182
H	0.124857	-5.678213	2.181158
H	0.233866	-4.184736	3.119041
H	-0.823159	-0.817302	2.281391
H	0.153486	-2.089285	3.030318
H	-1.626417	-2.101063	3.221982

TS(PhCF2●+DMA)

C	1.248792	3.466096	0.030275
C	0.506944	3.957349	-1.040000
C	1.358629	2.095421	0.232677
C	0.727990	1.211616	-0.646537

C	-0.024923	1.703365	-1.716595
C	-0.126422	3.073729	-1.911700
H	1.747630	4.151785	0.708757
H	0.420599	5.028984	-1.194703
H	1.941613	1.710472	1.062522
H	-0.535091	1.006581	-2.371346
H	-0.709064	3.455365	-2.744774
C	0.809888	-0.253202	-0.434654
F	1.793707	-0.596533	0.444046
F	1.032125	-0.935232	-1.581629
C	-0.761060	-0.897251	0.202806
N	-0.425510	-2.282533	0.382181
O	-1.605884	-0.618259	-0.716353
C	-0.940484	-0.122489	1.527795
C	-0.933808	-3.207946	-0.605164
H	-1.970261	-3.514164	-0.382313
H	-0.299962	-4.100623	-0.635191
H	-0.951073	-2.721008	-1.583174
C	0.077155	-2.835889	1.620193
H	0.667523	-3.734317	1.407424
H	-0.737674	-3.122702	2.308318
H	0.725775	-2.125544	2.133876
H	-1.189706	0.906196	1.268005
H	-0.060999	-0.131318	2.175050
H	-1.786452	-0.567950	2.060209

TS(PhCF2−+DMA-deprotonation)

C	-1.610724	-0.826807	1.680315
C	-2.263375	-2.036447	1.938996
C	-2.479084	-2.919613	0.878211
C	-2.061994	-2.607535	-0.407814
C	-1.412526	-1.383855	-0.681442
C	-1.189947	-0.501944	0.400136
H	-0.671172	0.433833	0.213575
H	-1.428189	-0.126548	2.492964
H	-2.586773	-2.288190	2.945039
H	-2.986920	-3.865478	1.057923
H	-2.234452	-3.303628	-1.223231
C	-0.792738	-1.125504	-1.965479
F	-0.838857	0.228261	-2.312059
F	-1.433155	-1.809932	-3.008167
C	1.990349	-1.672063	-2.165506
C	2.645223	-1.250217	-0.936608
H	2.026992	-2.741031	-2.390597
H	2.336917	-1.070416	-3.006215
H	0.634384	-1.436670	-2.030043
O	3.246910	-0.169411	-0.810285
N	2.541396	-2.098860	0.187882
C	1.498515	-3.104602	0.310943
H	0.619891	-2.718434	0.855188
H	1.881581	-3.978959	0.859263
H	1.154004	-3.435791	-0.667821
C	2.945737	-1.547900	1.467578
H	3.255866	-2.362165	2.137709
H	2.118637	-1.000509	1.957459
H	3.773360	-0.852847	1.31656

S5 Experimental Procedures and Analytical Data

2,2-Difluoro-2-phenylethanol (2a)

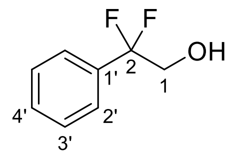

The title compound was prepared according to **GP2** using (trifluoromethyl)benzene (131.5 mg, 0.9 mmol) and purified by flash column chromatography (L = 13 cm, d = 2 cm; SiO_2, Cy:EA 9:1 → 5:1). **2a** was obtained as a colorless oil (**C_{gr}** 88.2 mg, 65% or **BDD** 111.0 mg, 78%).

18 mL scale: 226.2 mg, 80%.

90 mL scale: 1.12 g, 79%.

^{1}H NMR (400 MHz, $CDCl_3$) δ [ppm]: 7.55 – 7.50 (m, 2H, *H*-2'), 7.49 – 7.43 (m, 3H, *H*-3', *H*-4'), 3.95 (t, *J* = 13.4 Hz, 2H, *H*-1), 2.35 (s, 1H, 1-O*H*).

^{13}C NMR (101 MHz, $CDCl_3$) δ [ppm]: 134.5 (t, *J* = 25.5 Hz), 130.4 (t, *J* = 1.8 Hz), 128.7, 125.6 (t, *J* = 6.2 Hz), 120.7 (t, *J* = 243.6 Hz), 66.1 (t, *J* = 32.4 Hz).

^{19}F NMR (377 MHz, $CDCl_3$) δ [ppm]: -107.23 (t, *J* = 13.4 Hz).

The data matches that reported in the literature.[13]

2,2-Difluoro-2-(4-methylphenyl)ethanol (2b)

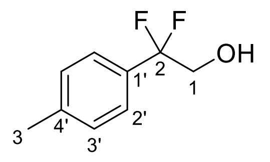

The title compound was prepared according to **GP2** using 4-methylbenzotrifluoride (158.5 mg, 0.9 mmol) and purified by flash column chromatography (L = 13 cm, d = 2 cm; SiO_2, pentanes:EA 9:1 → 5:1). **2b** was obtained as a colorless oil (**C_{gr}** 27.9 mg, 18% or **BDD** 77.3 mg, 50%).

^{1}H NMR (400 MHz, $CDCl_3$) δ [ppm]: 7.37 (d, *J* = 7.8 Hz, 2H, *H*-2'), 7.21 (d, *J* = 7.8 Hz, 2H, *H*-3'), 3.92 (td, *J* = 13.5 Hz, 7.0 Hz, 2H, *H*-1), 2.36 (s, 3H, *H*-3), 1.89 (t, *J* = 7.0 Hz, 1H, 1-O*H*).

^{13}C NMR (101 MHz, $CDCl_3$) δ [ppm]: 140.6, 131.6 (t, *J* = 25.7 Hz), 129.4, 125.5 (t, *J* = 6.1 Hz), 120.9 (t, *J* = 243.4 Hz), 66.2 (t, *J* = 32.7 Hz), 21.4.

^{19}F NMR (377 MHz, $CDCl_3$) δ [ppm]: -106.77 (t, *J* = 13.5 Hz, 2F).

The data matches that reported in the literature.[14]

2,2-Difluoro-2-(4-methoxyphenyl)ethanol (2c)

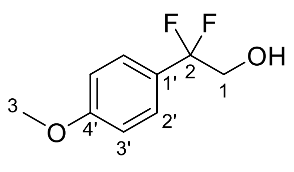

The title compound was prepared according to **GP2** using 4-methoxybenzotrifluoride (158.5 mg, 0.9 mmol) and purified by flash column chromatography (L = 13 cm, d = 2 cm; SiO_2, Cy:EA 9:1 → 5:1). **2c** was obtained as a colorless oil (**C$_{gr}$** 88.2 mg, 65% or **BDD** 99.7 mg, 78%).

^{1}H NMR (400 MHz, $CDCl_3$) δ [ppm]: 7.47 – 7.41 (m, 2H, *H*-2'), 6.98 – 6.93 (m, 2H, *H*-3'), 3.95 (t, J = 13.4 Hz, 2H, *H*-1), 3.83 (s, 3H, *H*-3).

^{13}C NMR (101 MHz, $CDCl_3$) δ [ppm]: 161.1, 127.1 (t, J = 6.1 Hz), 126.6 (t, J = 26.1 Hz), 120.9 (t, J = 243.2 Hz), 114.0, 66.2 (t, J = 33.1 Hz), 55.5.

^{19}F NMR (377 MHz, $CDCl_3$) δ [ppm]: -105.64 (t, J = 13.4 Hz).

The data matches that reported in the literature.[13]

2,2-Difluoro-2-(3-methoxyphenyl)ethanol (2d)

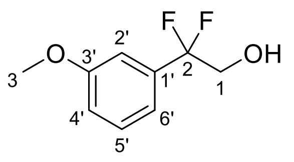

The title compound was prepared according to **GP2** using 3-methoxybenzotrifluoride (158.5 mg, 0.9 mmol) and purified by flash column chromatography (L = 13 cm, d = 2 cm; SiO_2, pentanes:EA 1:0 → 98:2 → 95:5). **2d** was obtained as a colorless oil (**C$_{gr}$** 69.4 mg, 42% or **BDD** 134.3 mg, 79%).

^{1}H NMR (400 MHz, $CDCl_3$) δ [ppm]: 7.36 (t, *J* = 8.0 Hz, 1H, *H*-5'), 7.11 – 7.07 (m, 1H, *H*-6'), 7.04 (t, *J* = 2.1 Hz, 1H, *H*-2'), 7.01 – 6.97 (m, 1H, *H*-4'), 3.95 (t, *J* = 13.5 Hz, 2H, *H*-1), 3.83 (s, 3H, *H*-3), 2.17 (s, 1H,1-O*H*).

^{13}C NMR (101 MHz, $CDCl_3$) δ [ppm]: 159.7, 135.9 (t, J = 25.6 Hz), 129.9, 120.6 (t, J = 244.1 Hz), 117.8 (t, J = 6.2 Hz), 116.0, 111.2 (t, J = 6.5 Hz), 66.1 (t, J = 32.3 Hz), 55.5.

^{19}F NMR (377 MHz, $CDCl_3$) δ [ppm]: -107.14 (t, *J* = 13.5 Hz).

The data matches that reported in the literature.[15]

2,2-Difluoro-2-(2-methoxyphenyl)ethanol (2e)

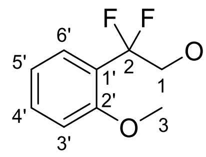

The title compound was prepared according to **GP2** using 2-methoxybenzotrifluoride (158.5 mg, 0.9 mmol) and purified by flash column chromatography (L = 13 cm, d = 2 cm; SiO_2, Cy:EA 9:1 → 5:1). **2e** was obtained as a colorless solid (**C$_{gr}$** 71.1 mg, 42% or **BDD** 99.7 mg, 59%).

^{1}H NMR (400 MHz, $CDCl_3$) δ [ppm]: 7.56 (dd, *J* = 7.6 Hz, 1.7 Hz, 1H, *H*-6'), 7.46 – 7.40 (m, 1H, *H*-4'), 7.03 (t, *J* = 7.6 Hz, 1H, *H*-5'), 6.97 (d, *J* = 8.3 Hz, 1H, *H*-3'), 4.16 (t, *J* = 14.1 Hz, 2H, *H*-1), 3.87 (s, 3H, *H*-3), 2.04 (s, 1H, 1-O*H*).

^{13}C NMR (101 MHz, $CDCl_3$) δ [ppm]: 157.0, 132.1, 127.8 (t, *J* = 8.6 Hz), 122.3 (t, *J* = 24.6 Hz), 120.7, 120.7 (t, *J* = 243.7 Hz), 111.9, 64.9 (t, *J* = 30.6 Hz), 55.9.

^{19}F NMR (377 MHz, $CDCl_3$) δ [ppm]: -105.40 (t, *J* = 14.1 Hz).

HRMS (APCI): calculated for $C_9H_{10}F_2O_2$ 188.0649; found 188.0643.

5-(1,1-Difluoro-2-hydroxyethyl)-2-methoxypyridine (2f)

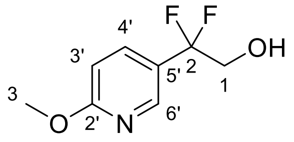

The title compound was prepared according to **GP2** using 2-methoxy-5-trifluoromethylpyridine (159.4 mg, 0.9 mmol). During work-up the pH was adjusted with sodium carbonate solution to pH = 10 before extraction. The product was purified by flash column chromatography (L = 17 cm, d = 2 cm; SiO_2, Cy:EA 9:1 → 3:1). **2f** was obtained as a beige solid (**C$_{gr}$** n.d. or **BDD** 74.9 mg, 44%).

^{1}H NMR (400 MHz, $CDCl_3$) δ [ppm]: 8.32 (s, 1H, *H*-6'), 7.70 (dd, *J* = 8.7, 2.5 Hz, 1H, *H*-4'), 6.79 (d, *J* = 8.7 Hz, 1H, *H*-3'), 4.02 – 3.91 (m, 5H, *H*-1, *H*-3), 2.32 (s, 1H, 1-O*H*).

^{13}C NMR (101 MHz, $CDCl_3$) δ [ppm]: 165.4, 145.0, 136.3, 123.6 (t, J = 26.5 Hz), 120.3 (t, J = 243.4 Hz), 65.9 (t, J = 33.3 Hz), 54.0.

^{19}F NMR (377 MHz, $CDCl_3$) δ [ppm]: -105.58 (t, *J* = 13.0 Hz).

HRMS (ESI+): $[M+H]^+$ calculated for $C_8H_9F_2NO_2$ 190.0674; found 190.0669.

2,2-Difluoro-2-(2-phenoxyphenyl)ethanol (2g)

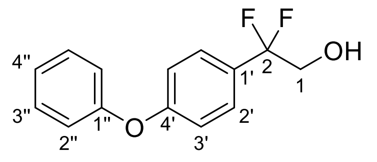

The title compound was prepared according to **GP2** using 4-phenoxybenzotrifluoride (214.4 mg, 0.9 mmol) and purified by flash column chromatography (L = 13 cm, d = 2 cm; SiO_2, Cy:EA 9:1 → 5:1). **2g** was obtained as a colorless solid (**C_{gr}** 164.4 mg, 73% or **BDD** 121.6 mg, 54%).

^{1}H NMR (400 MHz, $CDCl_3$) δ [ppm]: 7.50 – 7.45 (m, 2H, *H*-2'), 7.42 – 7.34 (m, 2H, *H*-2''), 7.21 – 7.14 (m, 1H, *H*-4''), 7.08 – 7.00 (m, 4H, *H*-3', *H*-3''), 3.97 (td, *J* = 13.3 Hz, 5.9 Hz, 2H, *H*-1, 2.22 (t, *J* = 6.0 Hz, 1H, 1-O*H*).

^{13}C NMR (101 MHz, $CDCl_3$) δ [ppm]: 159.4, 156.2, 130.1, 128.9 (t, *J* = 26.2 Hz), 127.4 (t, *J* = 6.1 Hz), 124.3, 120.7 (t, *J* = 243.5 Hz), 119.8, 118.2, 66.1 (t, *J* = 32.9 Hz).

^{19}F NMR (377 MHz, $CDCl_3$) δ [ppm]: -105.79 (t, *J* = 13.3 Hz).

HRMS (APCI): calculated for $C_{14}H_{12}F_2O_2$ 250.0805; found 250.0794.

2-([1,1'-Biphenyl]-4-yl)-2,2-difluoroethan-1-ol (2h)

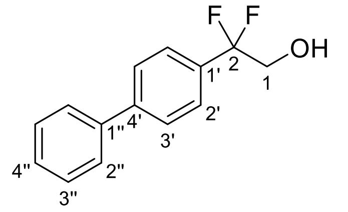

The title compound was prepared according to **GP2** using 4-(trifluoromethyl)-1,1'-biphenyl (200.0 mg, 0.9 mmol) and purified by flash column chromatography (L = 10 cm, d = 2 cm; SiO_2, Cy:EA 95:5 → 5:1). **2h** was obtained as a colorless solid (**C_{gr}**, 82.2 mg 39% or **BDD** 91.2 mg, 43%).

^{1}H NMR (400 MHz, $CDCl_3$) δ [ppm]: 7.67 (d, *J* = 8.1 Hz, 2H, *H*-2'), 7.62 – 7.58 (m, 4H, *H*-3', *H*-2''), 7.47 (t, *J* = 7.6 Hz, 2H, *H*-3''), 7.42 – 7.35 (m, 1H, *H*-4''), 4.02 (td, *J* = 13.3 Hz, 4.6 Hz, 2H, *H*-1), 1.99 (s, 1H, 1-O*H*).

^{13}C NMR (101 MHz, $CDCl_3$) δ [ppm]: 143.4, 140.3, 133.3 (t, J = 25.9 Hz), 129.1, 128.0, 127.5, 127.4, 126.1 (t, J = 6.1 Hz), 120.8 (t, J = 243.5 Hz), 66.2 (t, J = 32.7 Hz).

^{19}F NMR (377 MHz, $CDCl_3$) δ [ppm]: -106.83 (t, *J* = 13.3 Hz).

HRMS (APCI): calculated for $C_{14}H_{12}F_2O$ 234.0856; found 234.0851.

2,2-Difluoro-2-(naphthalen-1-yl)ethan-1-ol (2i)

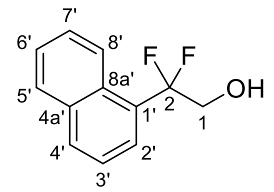

The title compound was prepared according to **GP2** using 1-(trifluoromethyl)naphthalene (176.6 mg, 0.9 mmol) and purified by flash column chromatography (L = 10 cm, d = 2 cm; SiO_2, Cy:EA 7:1 → 5:1). **2i** was obtained as a yellow oil (**C$_{gr}$** 50.6 mg, 27% or **BDD** 76.4 mg, 41%).

^{1}H NMR (400 MHz, $CDCl_3$) δ [ppm]: 8.22 (dd, *J* = 8.3 Hz, 1.9 Hz, 1H, *H*-8'), 7.96 (d, *J* = 8.2 Hz, 1H, *H*-5'), 7.91 (dd, *J* = 8.2 Hz, 1.5 Hz, 1H, *H*-4'), 7.78 (d, *J* = 7.3 Hz, 1H, *H*-2'), 7.61 – 7.48 (m, 3H, *H*-3', *H*-6', *H*-7'), 4.23 (t, *J* = 13.7 Hz, 2H, *H*-1), 2.16 (s, 1H, 1-O*H*).

^{13}C NMR (101 MHz, $CDCl_3$) δ [ppm]: 134.2, 131.7, 129.9 – 129.6 (m), 129.4, 129.2, 127.2, 126.2, 125.5 (t, *J* = 9.1 Hz), 124.7, 124.6 (t, *J* = 4.8 Hz), 122.0 (t, *J* = 244.5 Hz), 66.0 (t, *J* = 30.6 Hz).

^{19}F NMR (377 MHz, $CDCl_3$) δ [ppm]: -102.15 (t, *J* = 13.7 Hz).

The data matches that reported in the literature.[16]

2,2-Difluoro-2-(4-fluorophenyl)ethanol (2j)

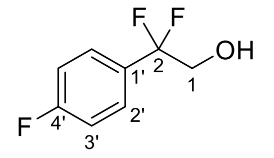

The title compound was prepared according to **GP2** using 4-fluorobenzotrifluoride (147.7 mg, 0.9 mmol) and purified by flash column chromatography (L = 17 cm, d = 2 cm; SiO_2, pentane:diethyl ether 95:5 → 5:1). **2j** was obtained as a yellowish oil (**C$_{gr}$** 54.8 mg, 35% or **BDD** 102.7 mg, 65%).

^{1}H NMR (400 MHz, $CDCl_3$) δ [ppm]: 7.50 (dd, *J* = 8.5 Hz, 5.2 Hz, 2H, *H*-2'), 7.12 (t, *J* = 8.5 Hz, 2H, *H*-3'), 3.93 (t, *J* = 13.3 Hz, 2H, *H*-1), 2.54 (broad, s, 1H, 1-O*H*).

^{13}C NMR (101 MHz, $CDCl_3$) δ [ppm]: 163.9 (d, *J* = 250.1 Hz), 130.6 (tq, *J* = 22.8, 3.6 Hz), 127.8 (dt, *J* = 8.7, 6.2 Hz), 120.4 (t, *J* = 243.8 Hz), 115.8 (d, *J* = 22.0 Hz), 65.9 (t, *J* = 32.7 Hz).

^{19}F NMR (377 MHz, $CDCl_3$) δ [ppm]: -106.00 (t, *J* = 13.2 Hz), -110.29 – -110.43 (m.

The data matches that reported in the literature.[13]

2,2-Difluoro-2-(3-fluorophenyl)ethanol (2k)

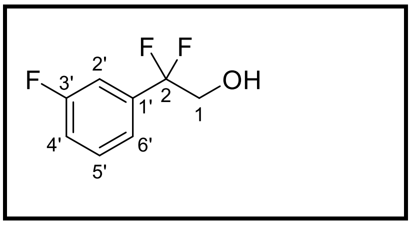

The title compound was prepared according to **GP2** using 3-fluorobenzotrifluoride (147.7 mg, 0.9 mmol) and purified by flash column chromatography (L = 17 cm, d = 2 cm; SiO_2, Cy:EA 95:5 → 5:1). **2k** was obtained as a yellowish oil (**C_{gr}** 46.4 mg, 29% or **BDD** 93.2 mg, 59%).

^{1}H NMR (400 MHz, $CDCl_3$) δ [ppm]: 7.47 – 7.40 (m, 1H, *H*-2'), 7.31 (d, *J* = 7.8 Hz, 1H, *H*-6'), 7.26 – 7.22 (m, 1H, *H*-4'), 7.21 – 7.14 (m, 1H, *H*-5'), 4.04 – 3.89 (m, 2H, *H*-1), 1.99 (s, 1H, 1-O*H*).

^{13}C NMR (101 MHz, $CDCl_3$) δ [ppm]: 162.7 (d, *J* = 247.4 Hz), 136.8 (td, *J* = 26.2, 7.3 Hz), 130.5 (d, *J* = 8.1 Hz), 121.4 (td, *J* = 6.2, 3.2 Hz), 120.0 (td, *J* = 244.3, 2.2 Hz), 117.6 (d, *J* = 1.8 Hz), 113.2 (dt, *J* = 23.6, 6.4 Hz), 65.9 (t, *J* = 32.4 Hz).

^{19}F NMR (377 MHz, $CDCl_3$) δ [ppm]: -106.93 (t, *J* = 13.3 Hz), -111.62 (td, *J* = 8.9, 5.7 Hz).

The data matches that reported in the literature.[17]

S6 Spectra

2,2-difluoro-2-phenylethanol (2a) ^{1}H NMR (400 MHz, $CDCl_3$)

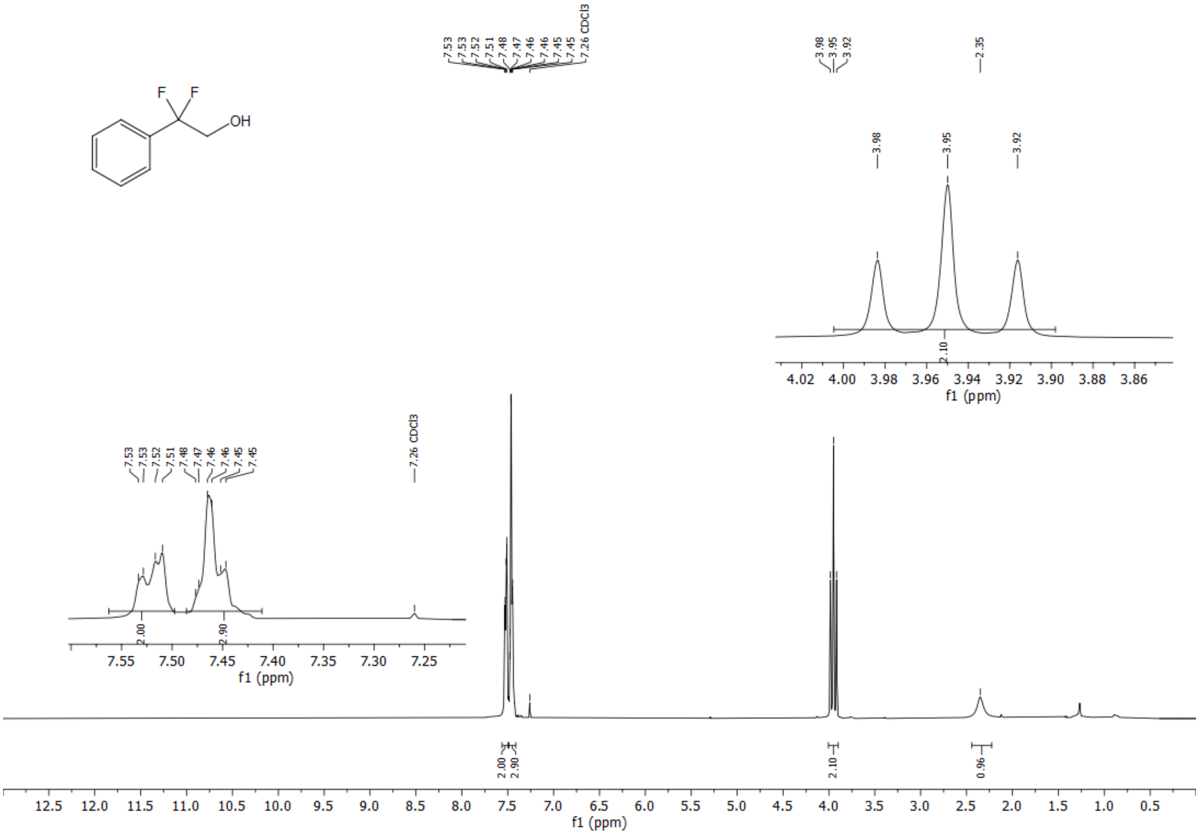

2,2-difluoro-2-phenylethanol (2a) ^{13}C NMR (100 MHz, $CDCl_3$)

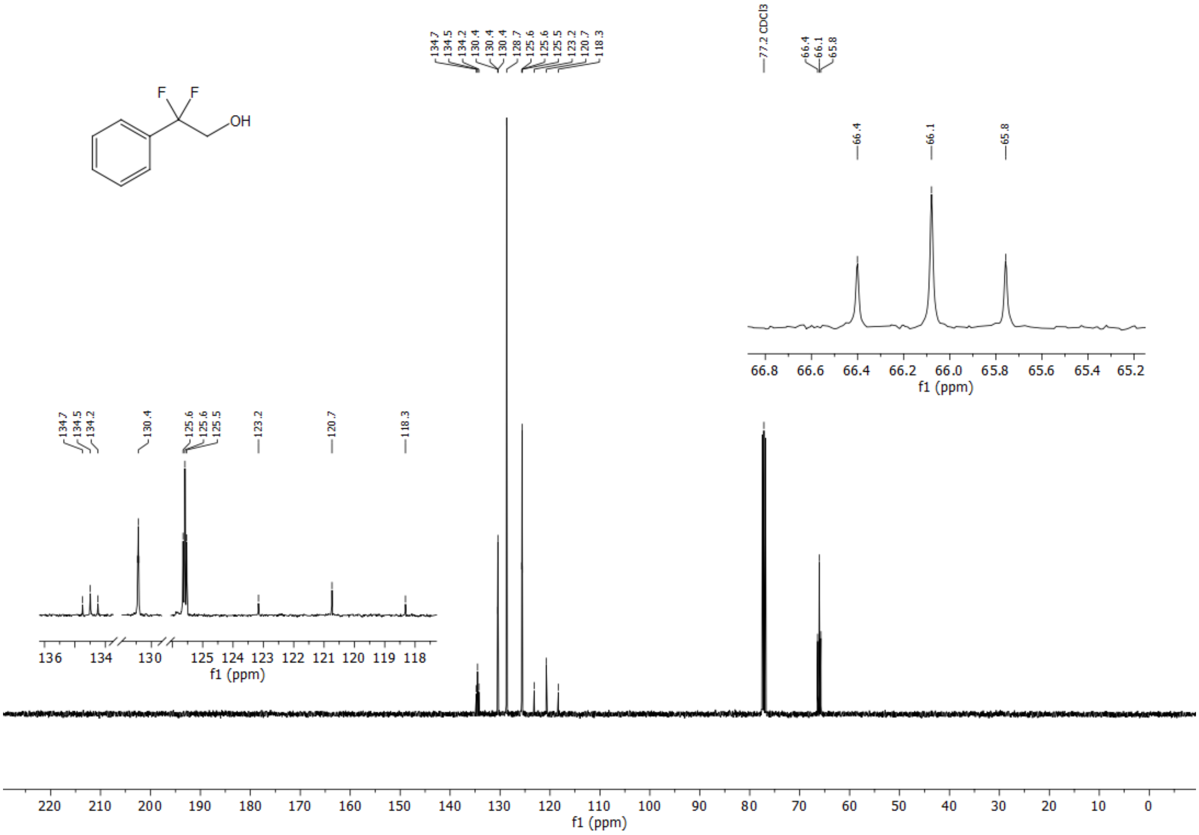

2,2-difluoro-2-phenylethanol (2a) ^{19}F NMR (377 MHz, $CDCl_3$)

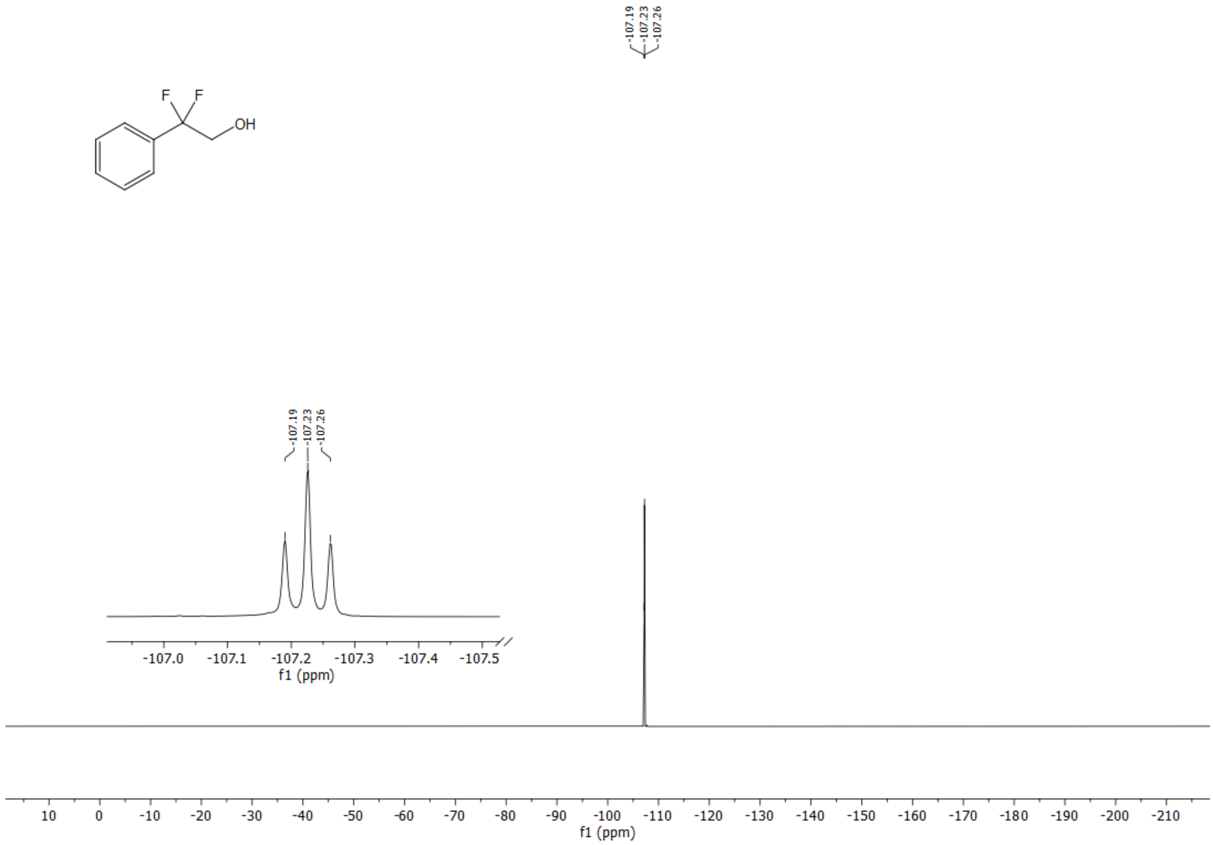

2,2-Difluoro-2-(4-methylphenyl)ethanol (2b) ^{1}H NMR (400 MHz, $CDCl_3$)

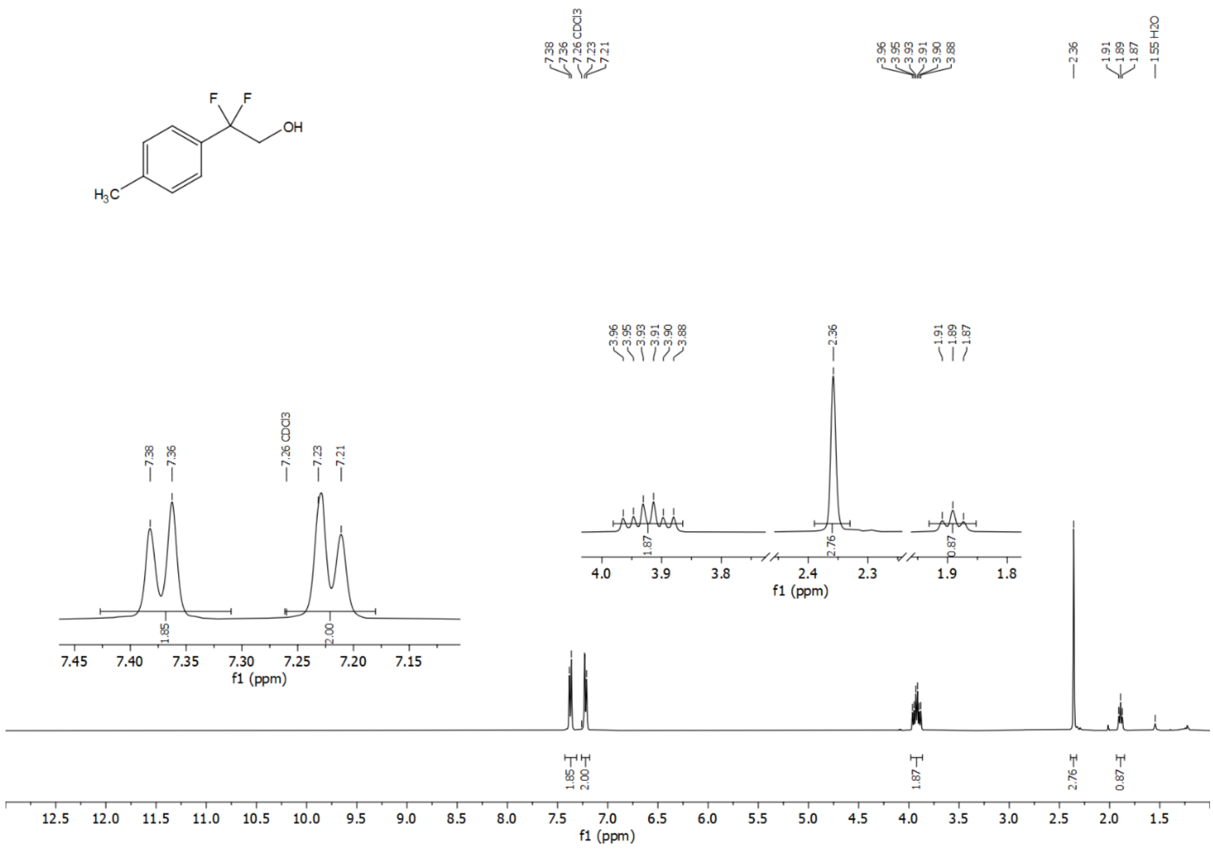

2,2-Difluoro-2-(4-methylphenyl)ethanol (2b) ^{13}C NMR (100 MHz, $CDCl_3$)

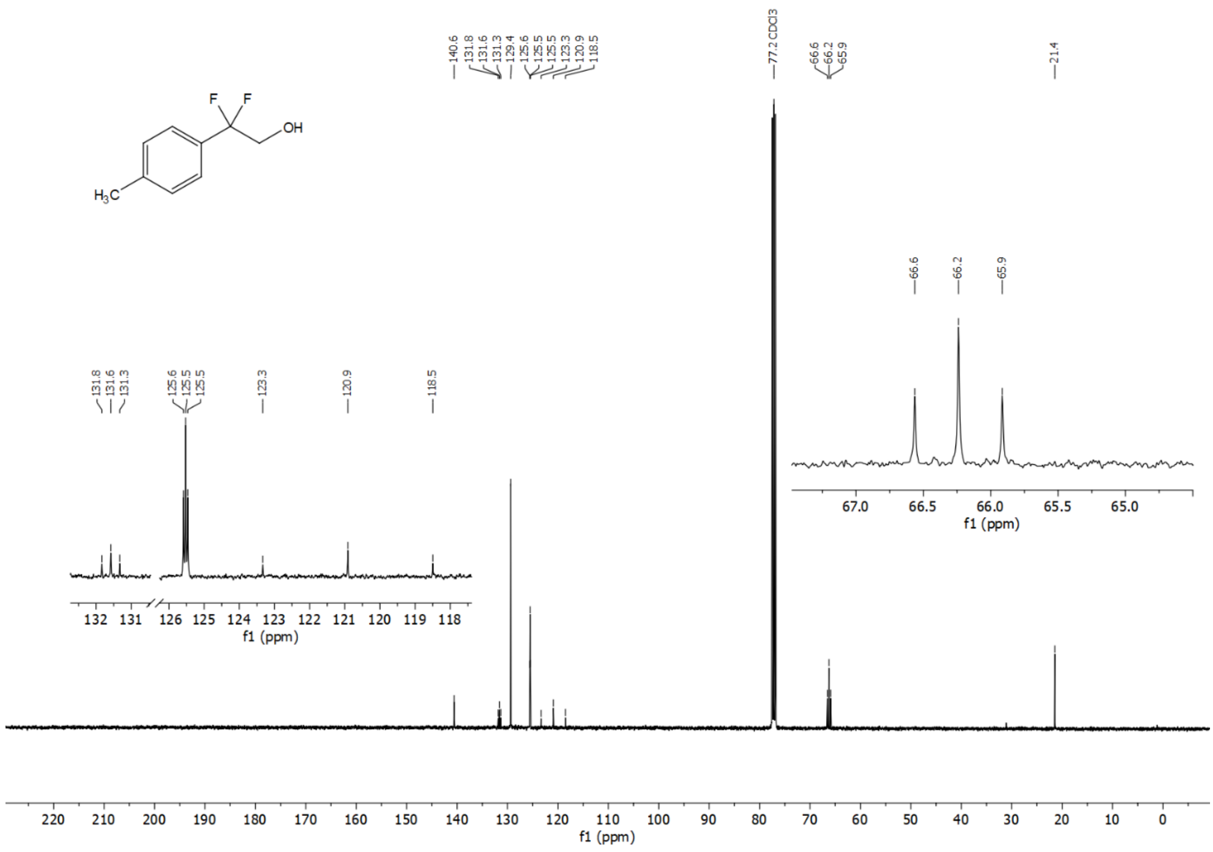

2,2-Difluoro-2-(4-methylphenyl)ethanol (2b) ^{19}F NMR (377 MHz, $CDCl_3$)

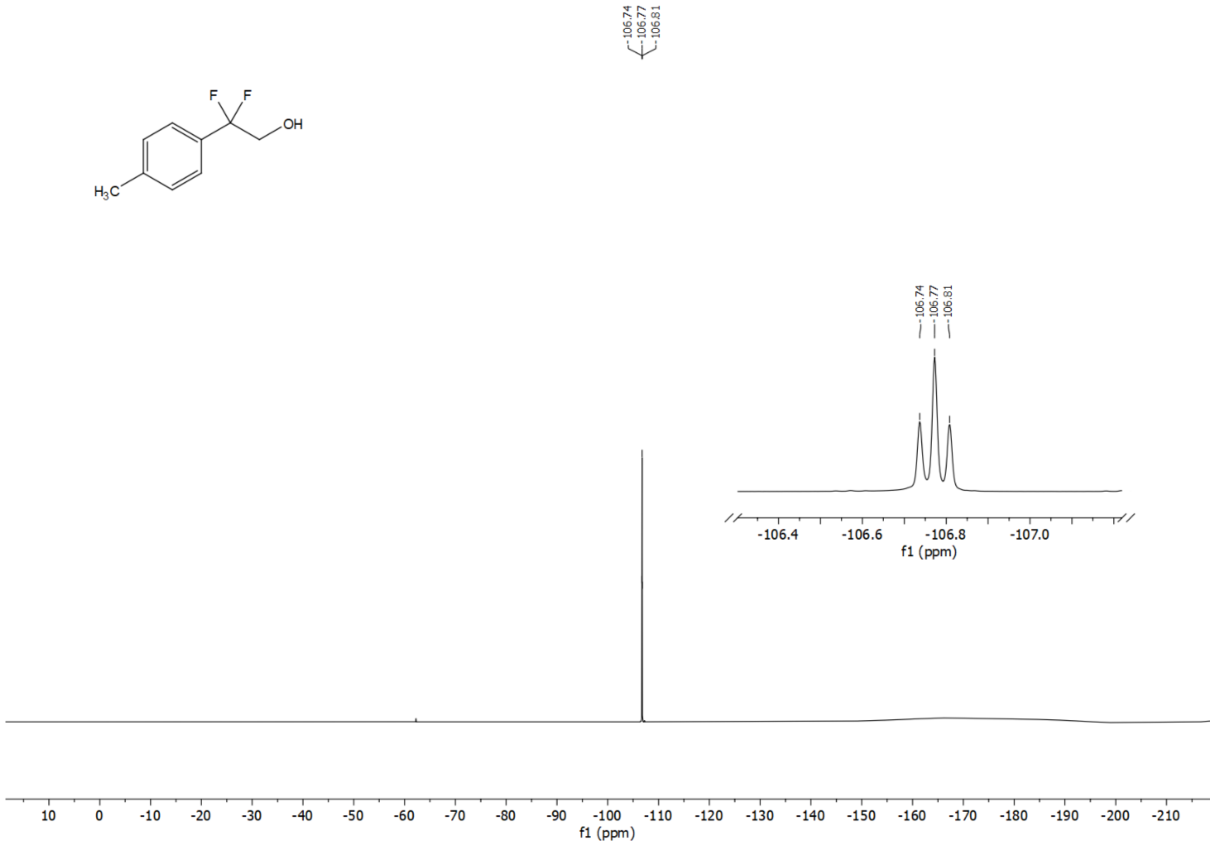

2,2-Difluoro-2-(4-methoxyphenyl)ethanol (2c) ^{1}H NMR (400 MHz, $CDCl_3$)

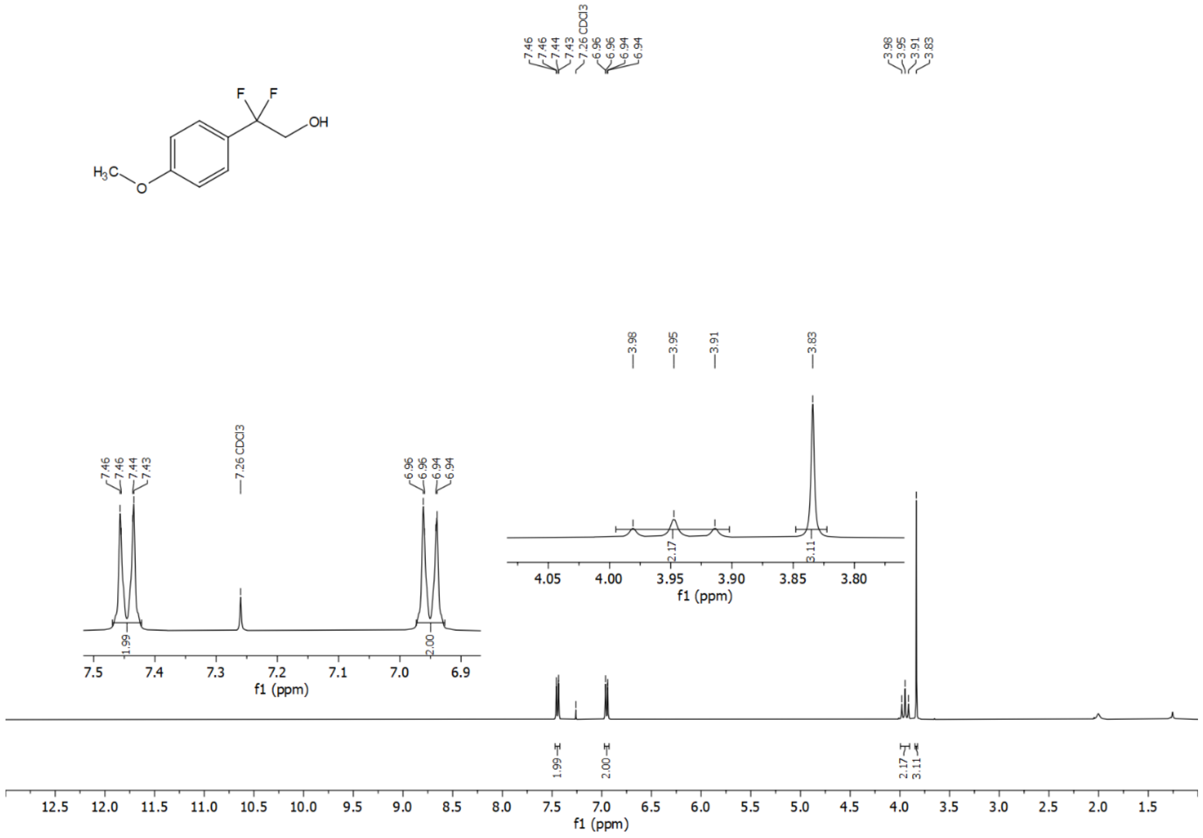

2,2-Difluoro-2-(4-methoxyphenyl)ethanol (2c) ^{13}C NMR (100 MHz, $CDCl_3$)

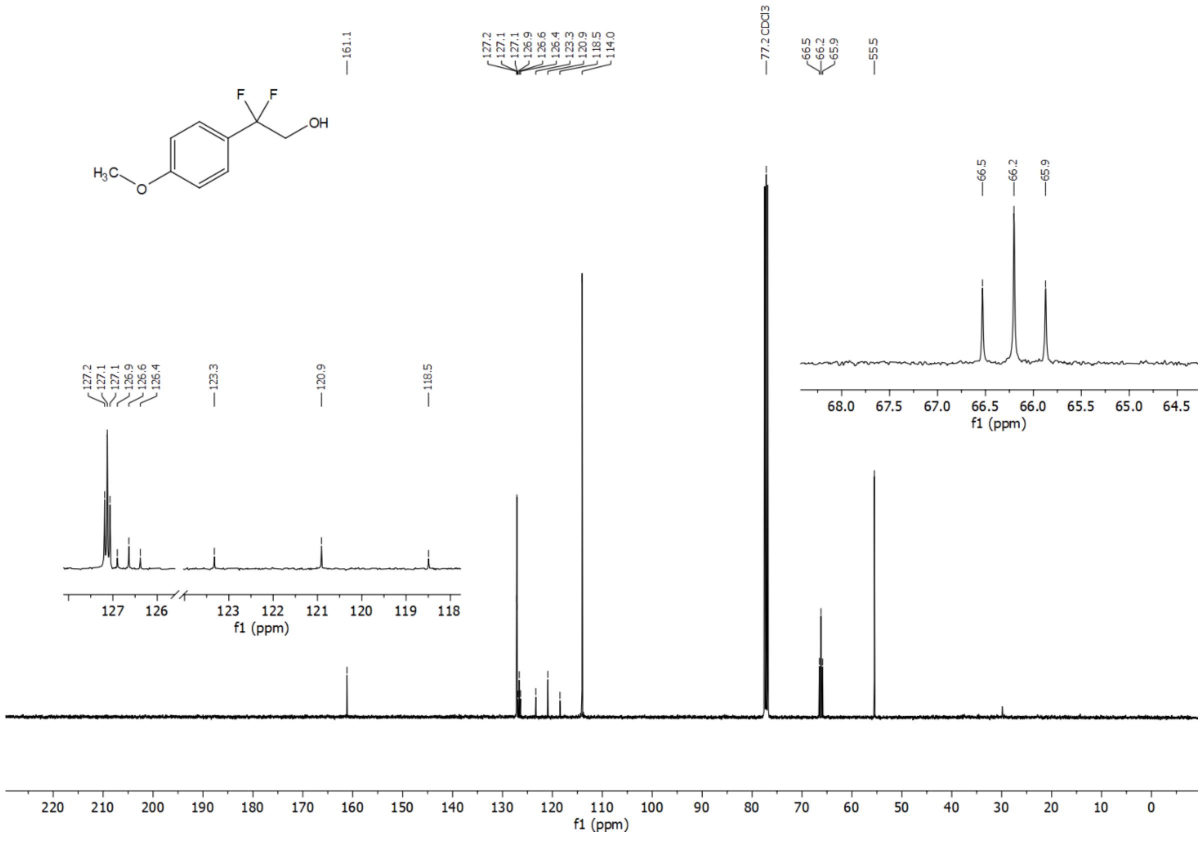

2,2-Difluoro-2-(4-methoxyphenyl)ethanol (2c) ^{19}F NMR (377 MHz, $CDCl_3$)

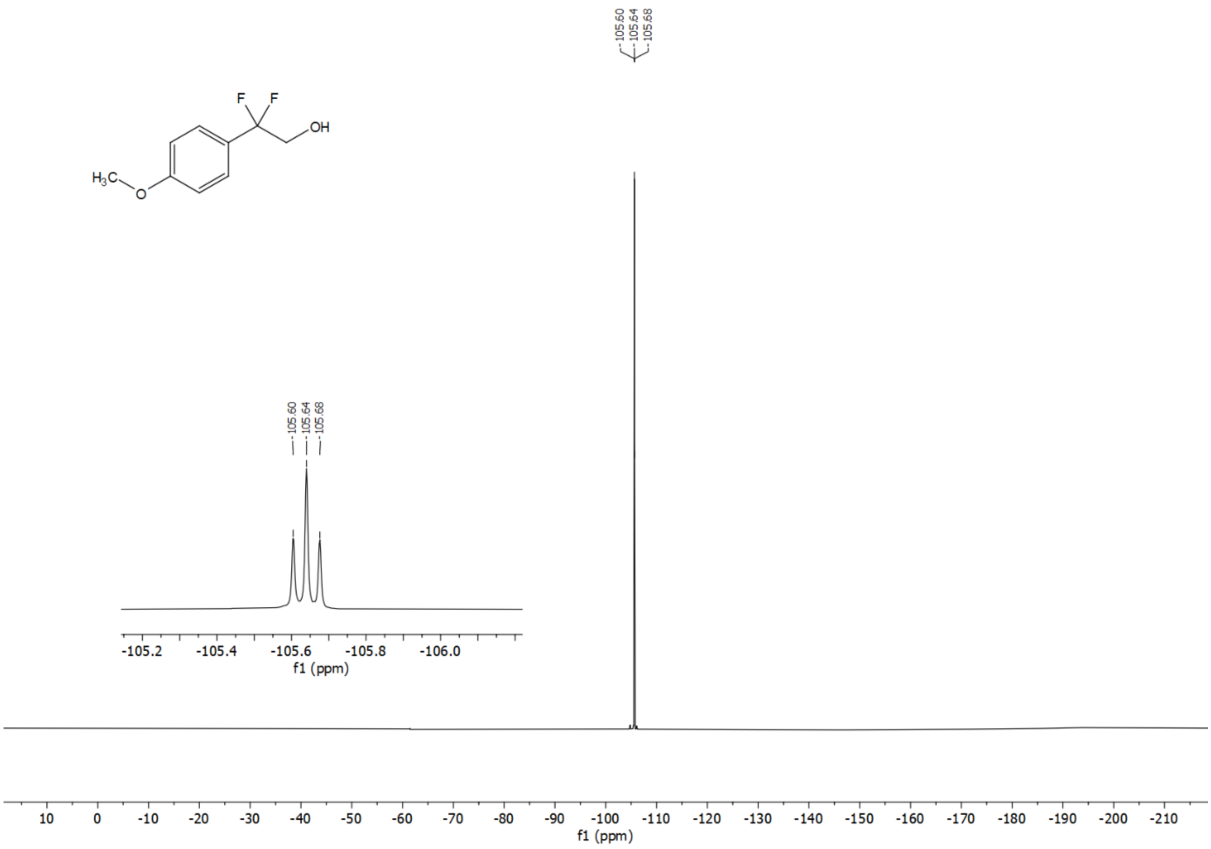

2,2-Difluoro-2-(3-methoxyphenyl)ethanol (2d) ^{1}H NMR (400 MHz, $CDCl_3$)

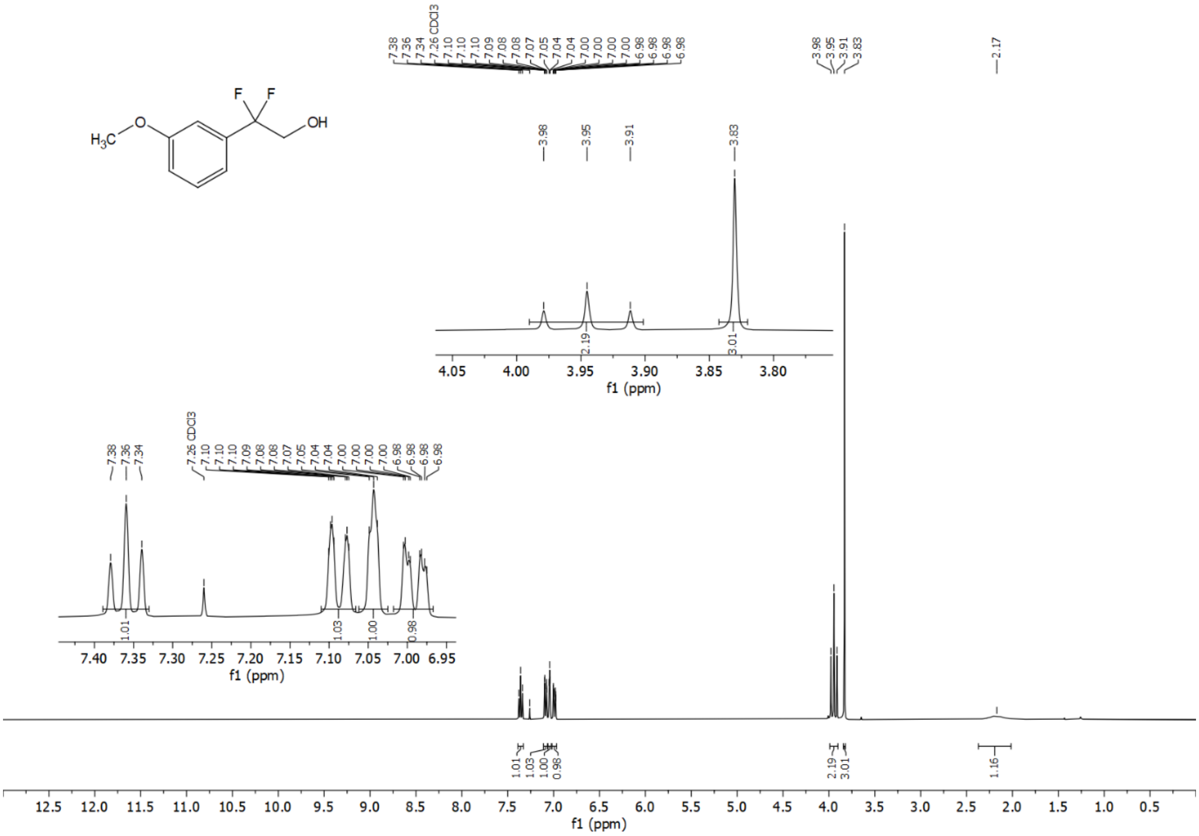

2,2-Difluoro-2-(3-methoxyphenyl)ethanol (2d) ^{13}C NMR (100 MHz, $CDCl_3$)

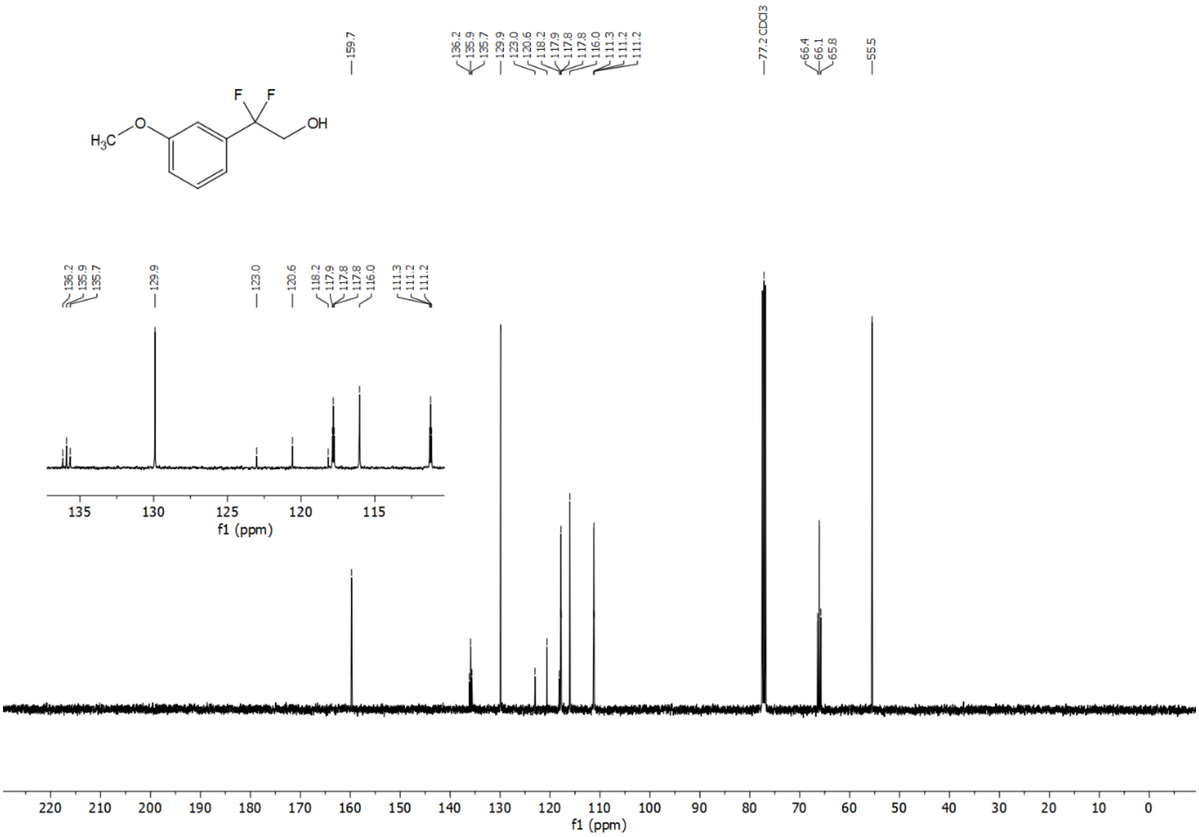

2,2-Difluoro-2-(3-methoxyphenyl)ethanol (2d) ^{19}F NMR (377 MHz, $CDCl_3$)

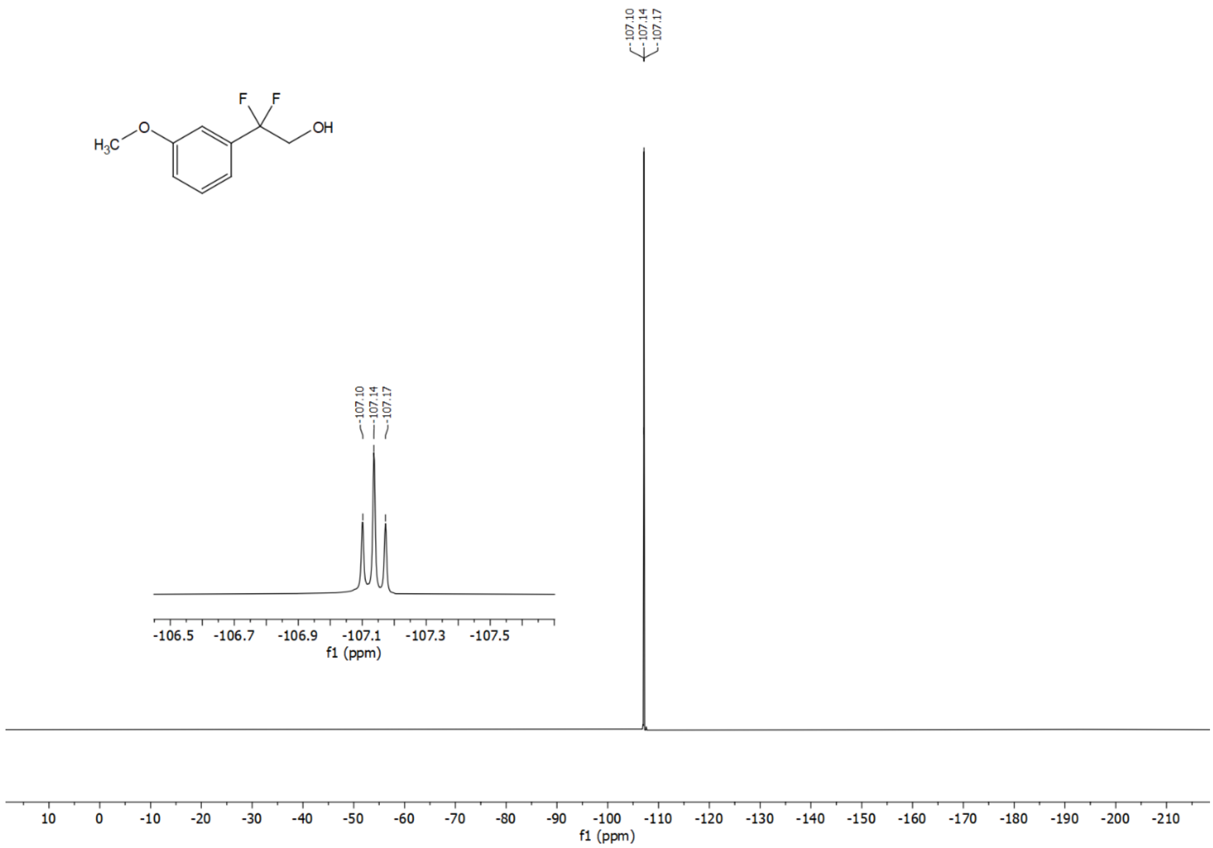

2,2-Difluoro-2-(2-methoxyphenyl)ethanol (2e) ^{1}H NMR (400 MHz, $CDCl_3$)

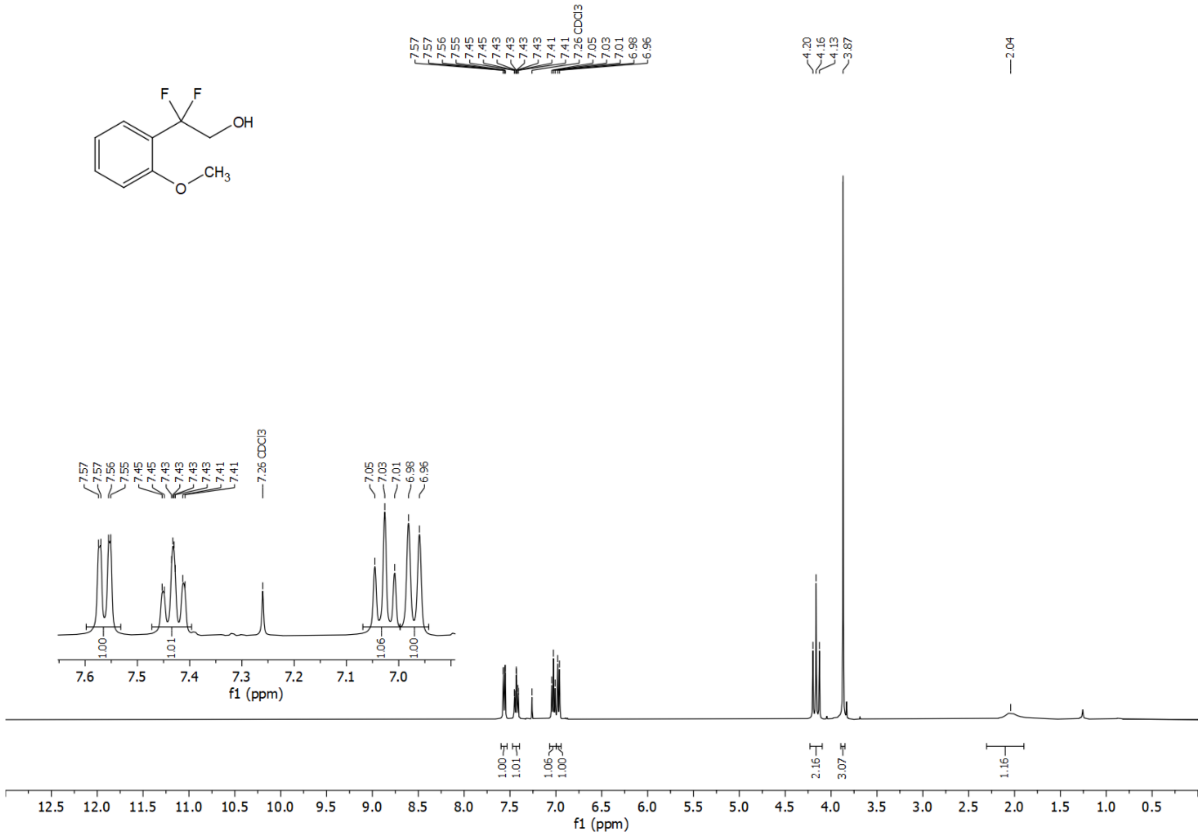

2,2-Difluoro-2-(2-methoxyphenyl)ethanol (2e) ^{13}C NMR (100 MHz, $CDCl_3$)

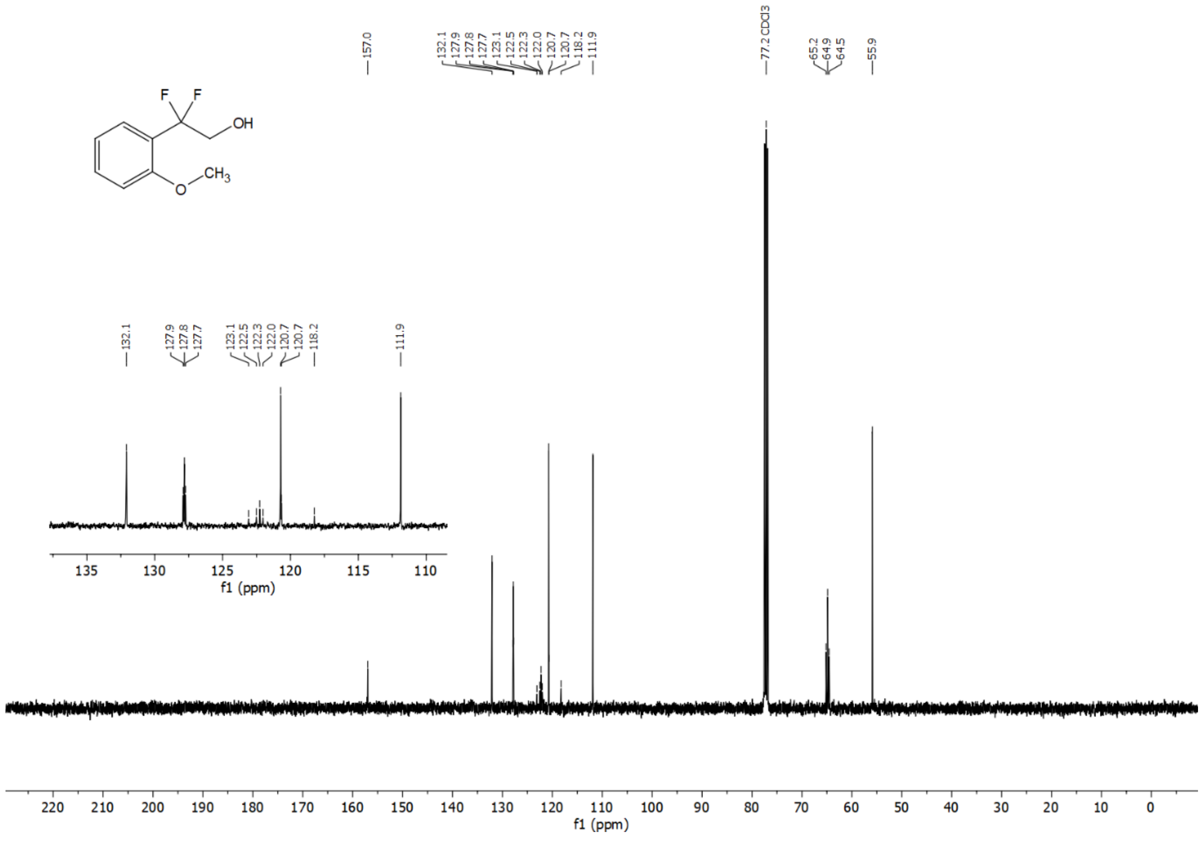

2,2-Difluoro-2-(2-methoxyphenyl)ethanol (2e) ^{19}F NMR (377 MHz, $CDCl_3$)

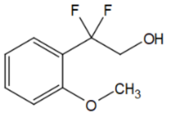

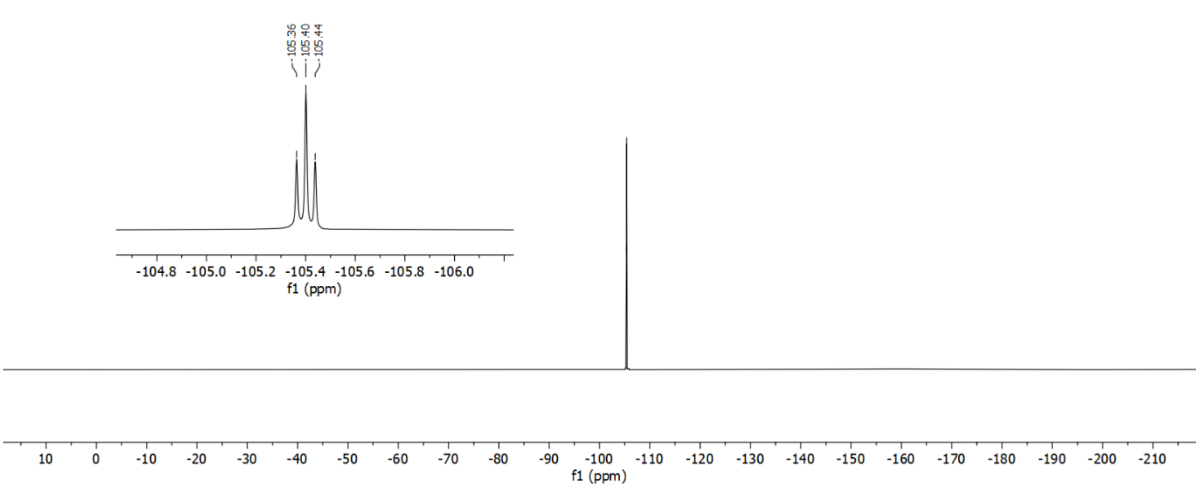

5-(1,1-Difluoro-2-hydroxyethyl)-2-methoxypyridine (2f) ^{1}H NMR (400 MHz, $CDCl_3$)

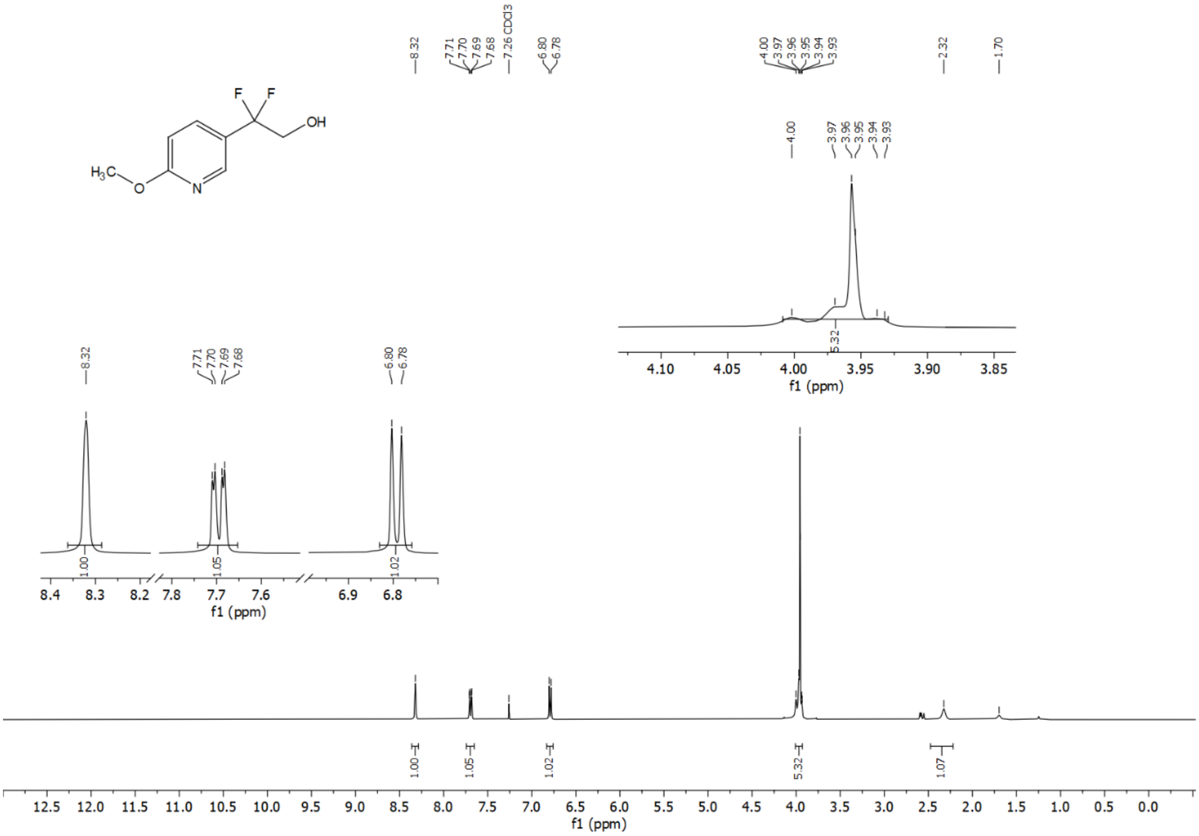

5-(1,1-Difluoro-2-hydroxyethyl)-2-methoxypyridine (2f) ^{13}C NMR (100 MHz, $CDCl_3$)

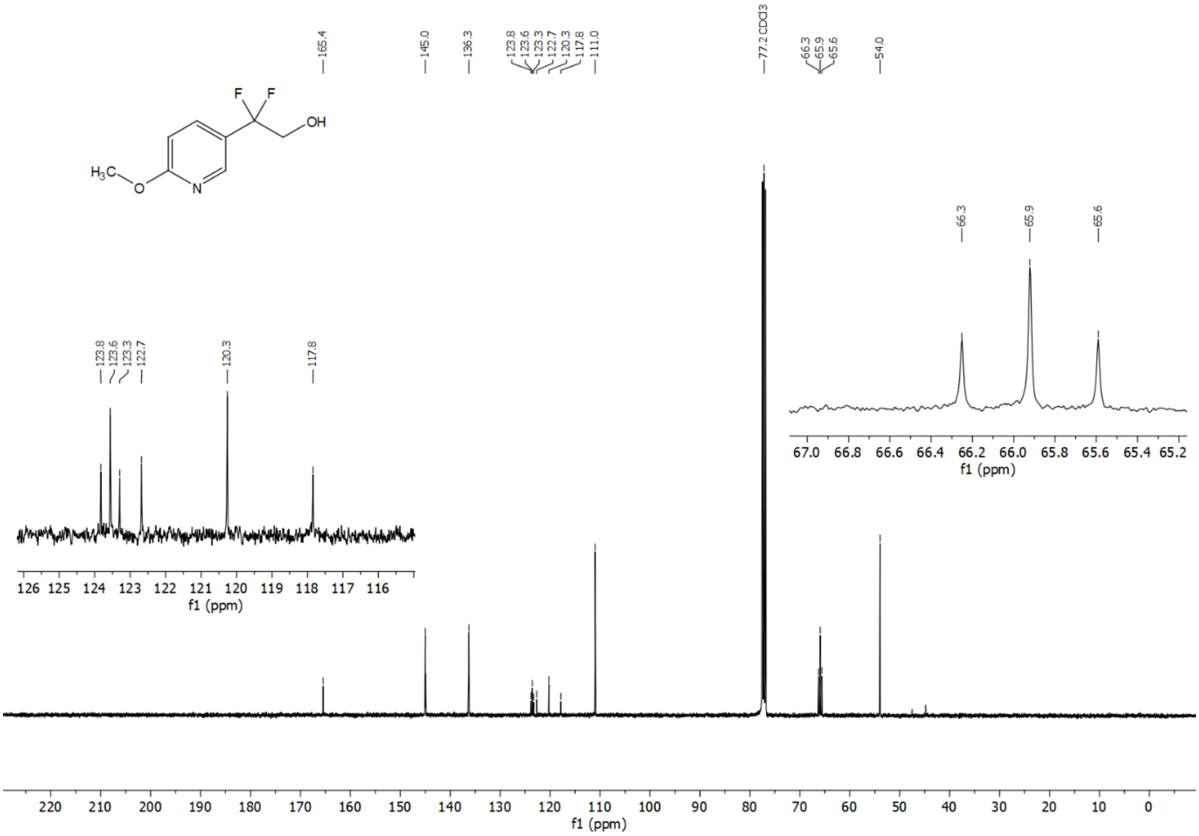

5-(1,1-Difluoro-2-hydroxyethyl)-2-methoxypyridine (2f) ^{19}F NMR (377 MHz, $CDCl_3$)

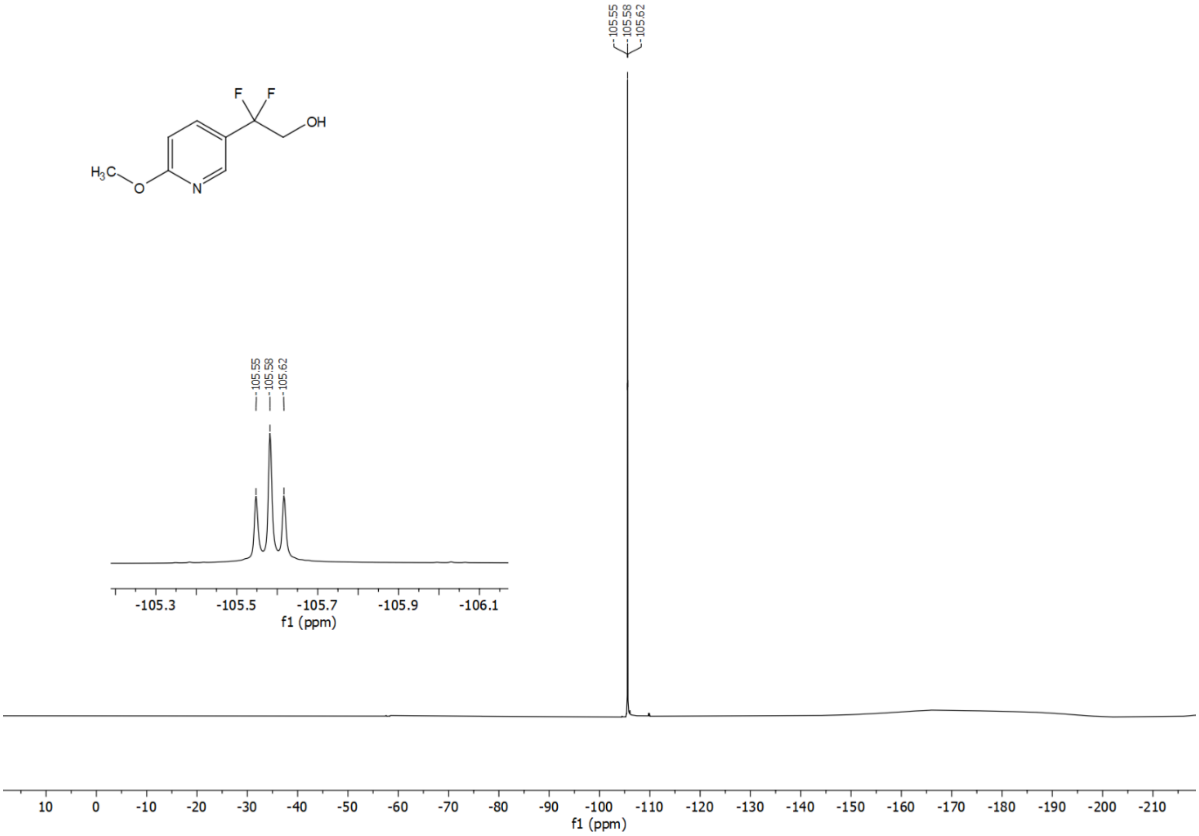

2,2-Difluoro-2-(4-phenoxyphenyl)ethanol (2g) ^{1}H NMR (400 MHz, CDCl$_3$)

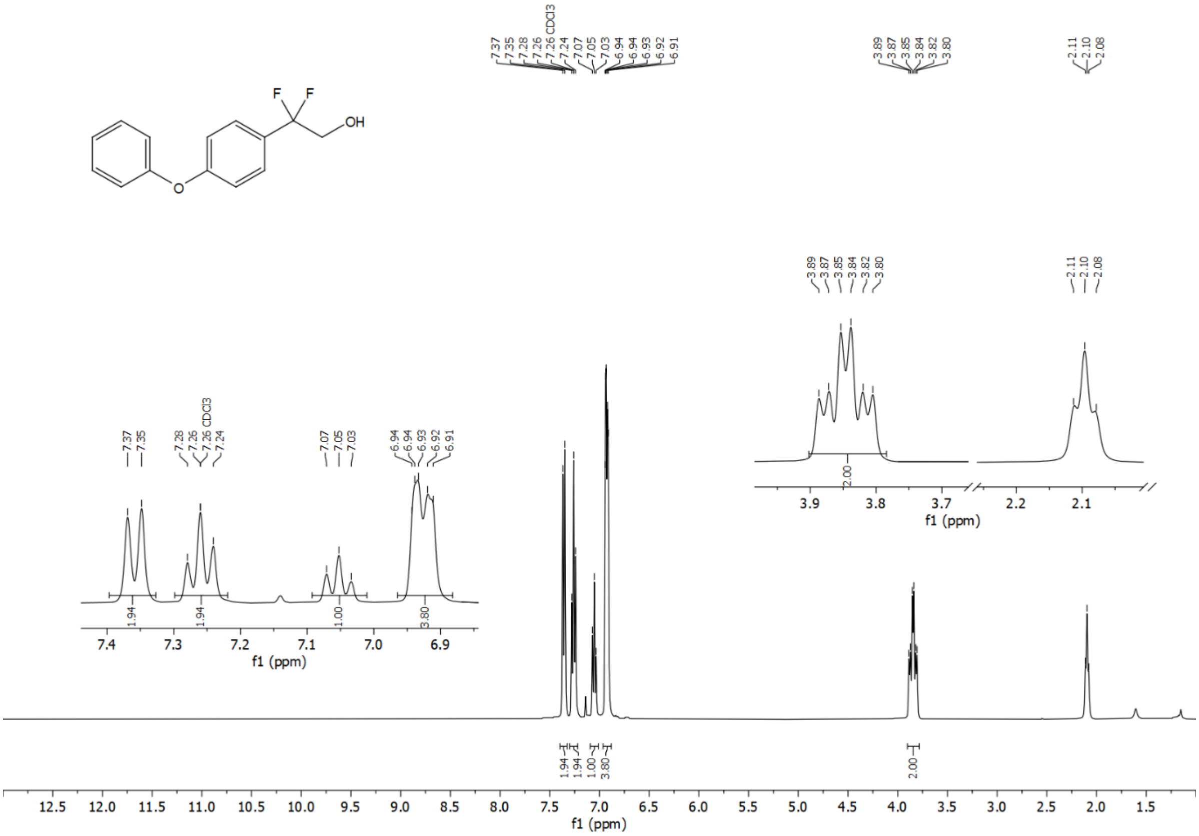

2,2-Difluoro-2-(4-phenoxyphenyl)ethanol (2g) ^{13}C NMR (100 MHz, CDCl$_3$)

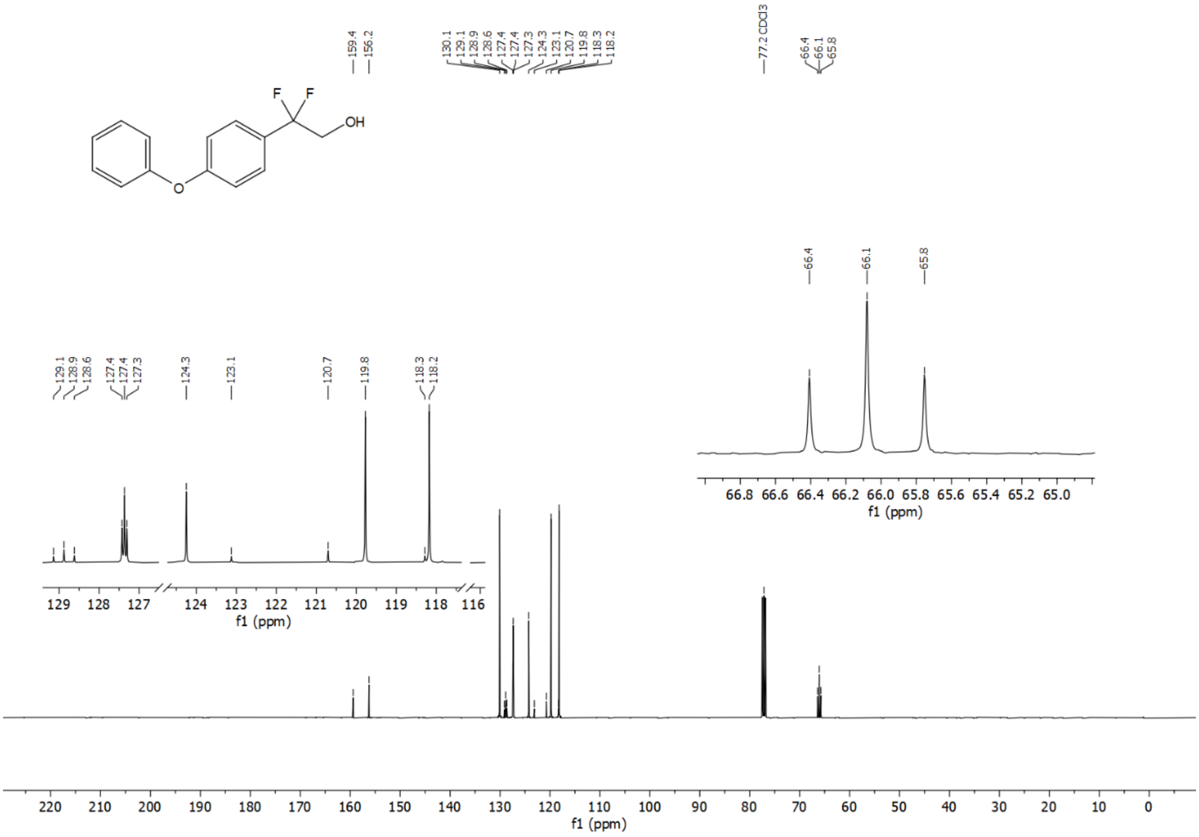

2,2-Difluoro-2-(4-phenoxyphenyl)ethanol (2g) ^{19}F NMR (377 MHz, $CDCl_3$)

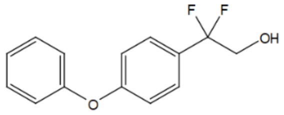

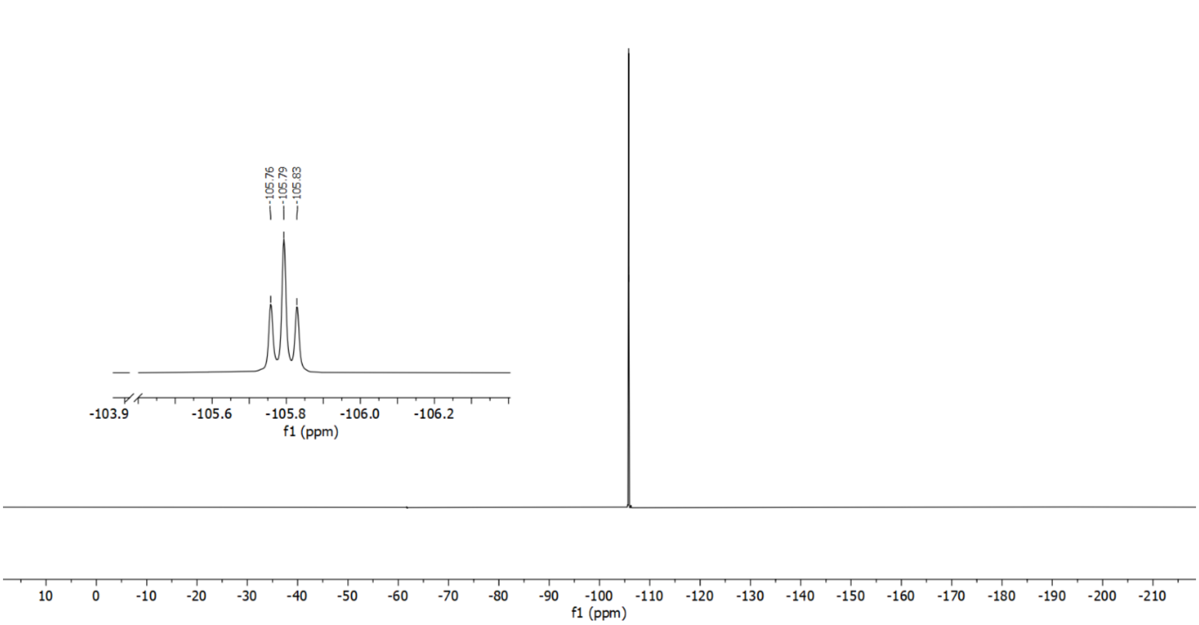

2-([1,1'-Biphenyl]-4-yl)-2,2-difluoroethan-1-ol (2h) ^{1}H NMR (400 MHz, $CDCl_3$)

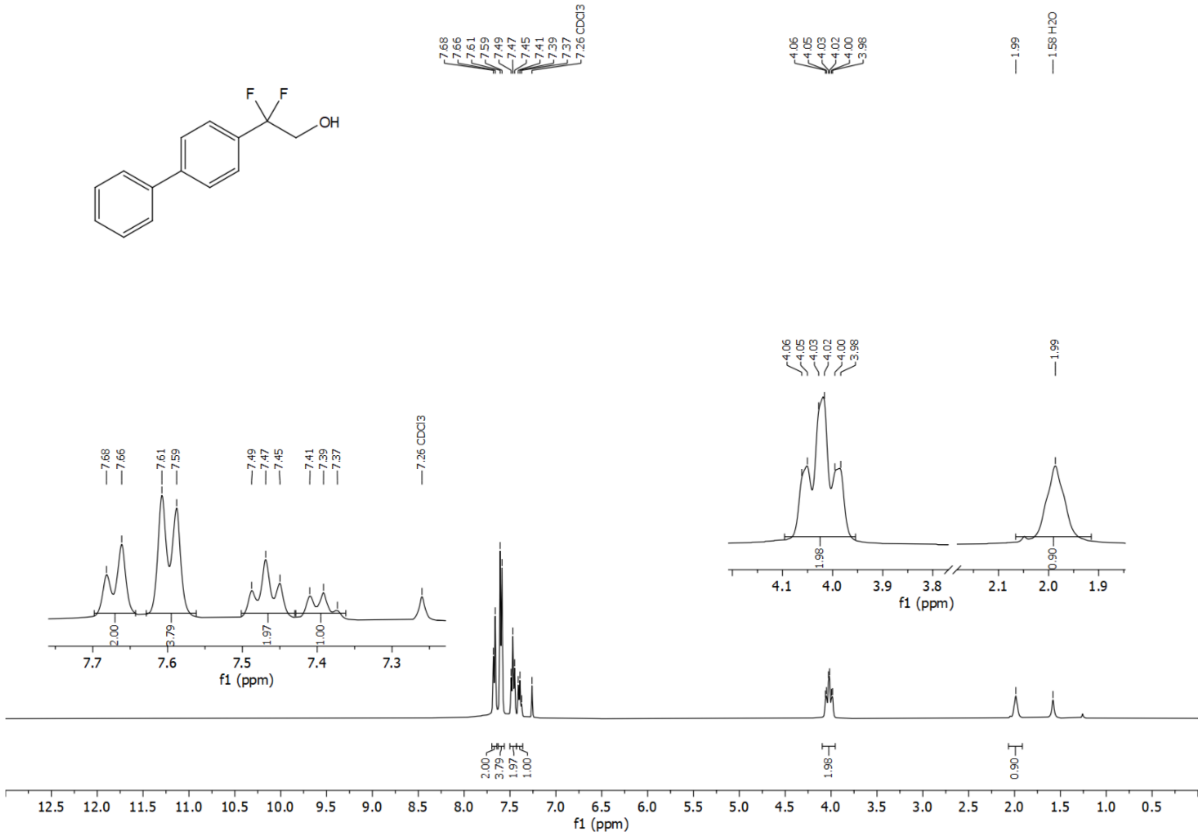

2-([1,1'-Biphenyl]-4-yl)-2,2-difluoroethan-1-ol (2h) ^{13}C NMR (100 MHz, $CDCl_3$)

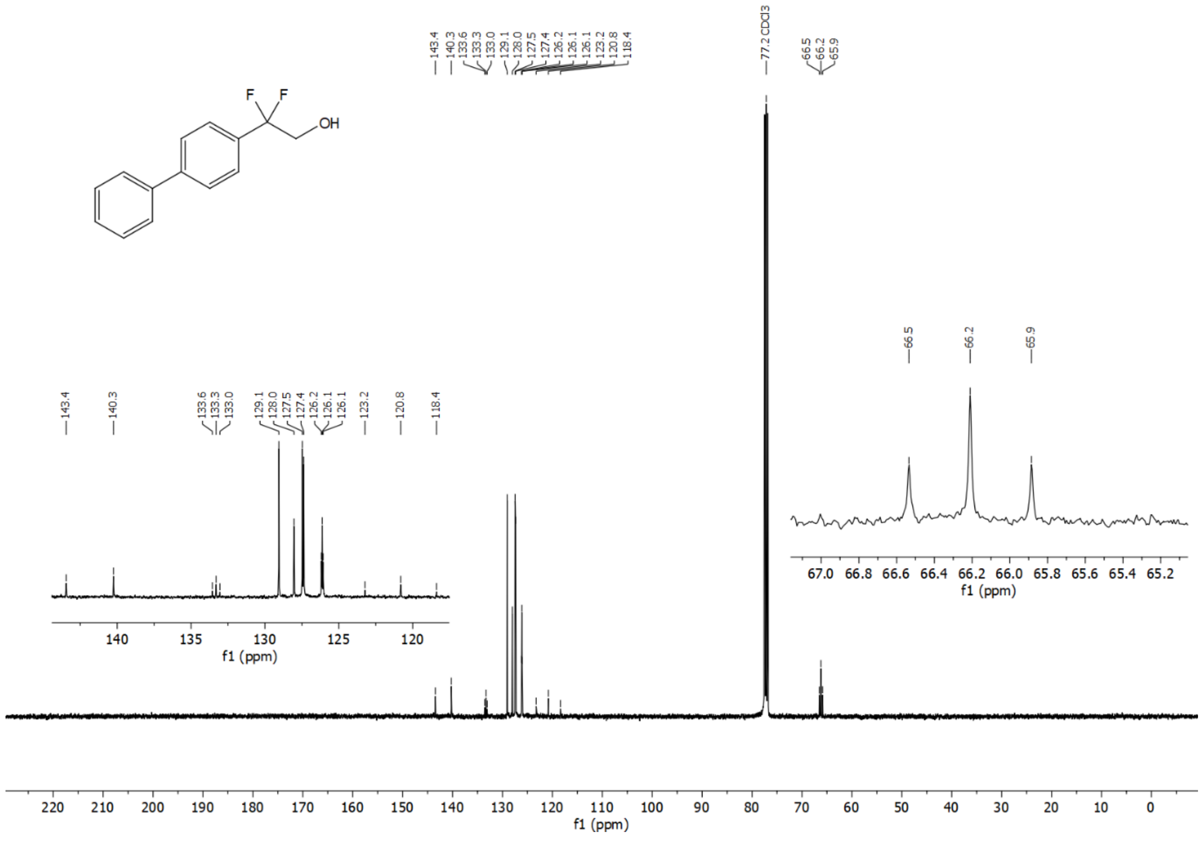

2-([1,1'-Biphenyl]-4-yl)-2,2-difluoroethan-1-ol (2h) ^{19}F NMR (377 MHz, $CDCl_3$)

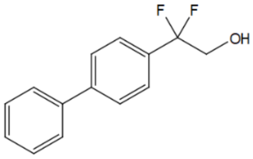

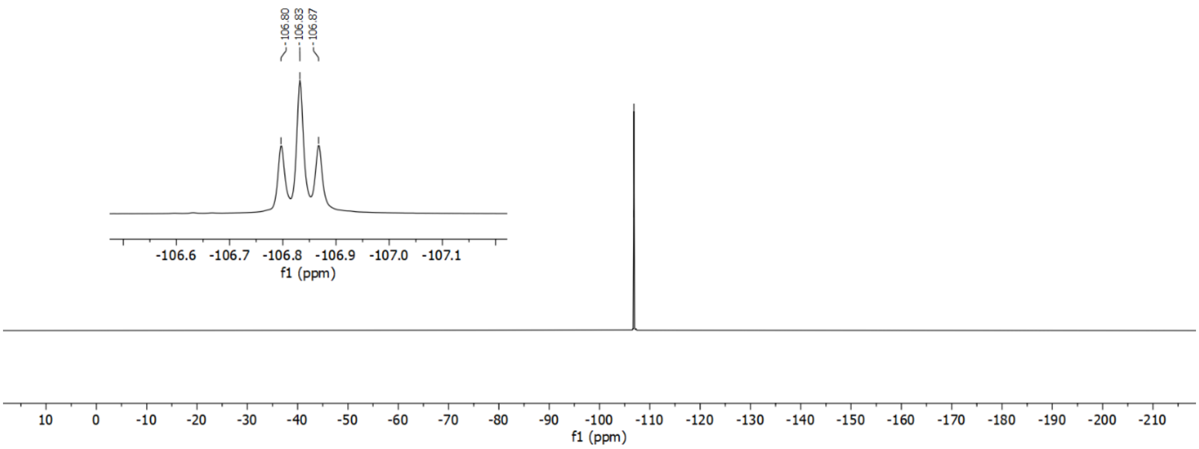

2,2-Difluoro-2-(naphthalen-1-yl)ethan-1-ol (2i) ^{1}H NMR (400 MHz, $CDCl_3$)

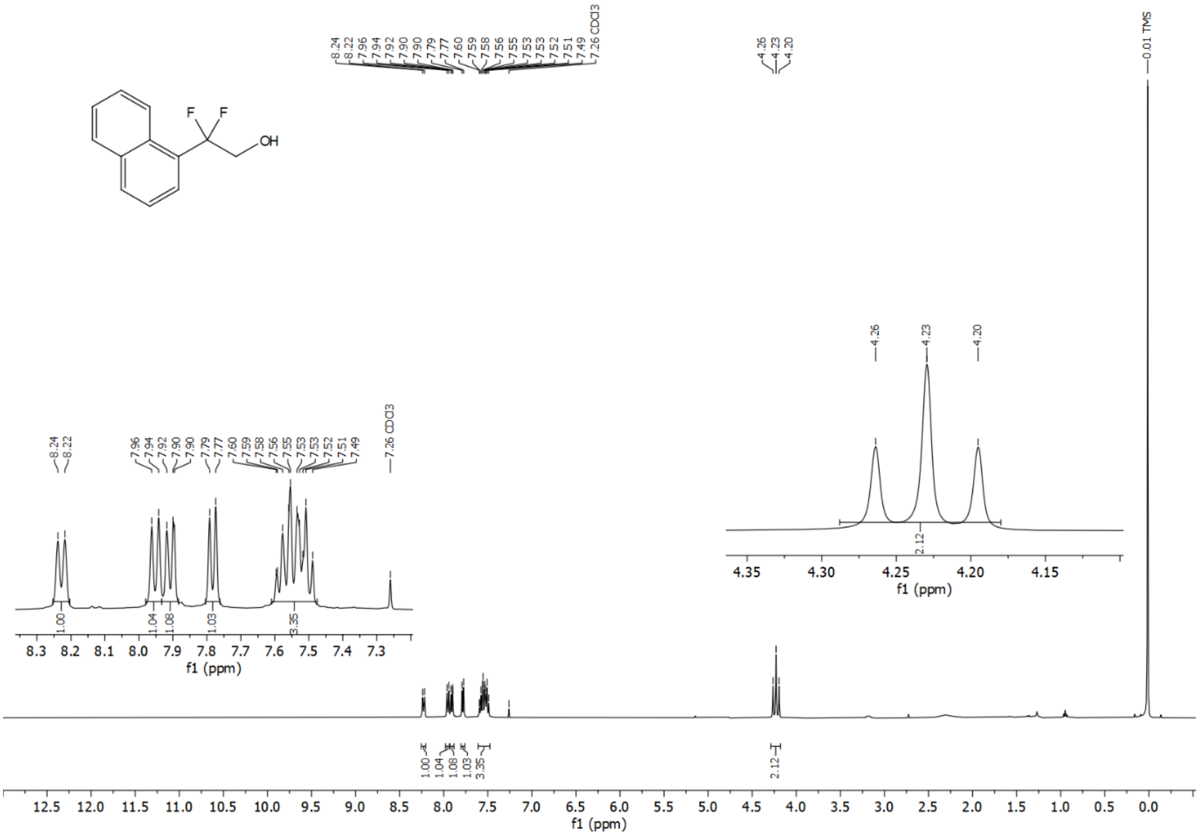

2,2-Difluoro-2-(naphthalen-1-yl)ethan-1-ol (2i) ^{13}C NMR (100 MHz, $CDCl_3$)

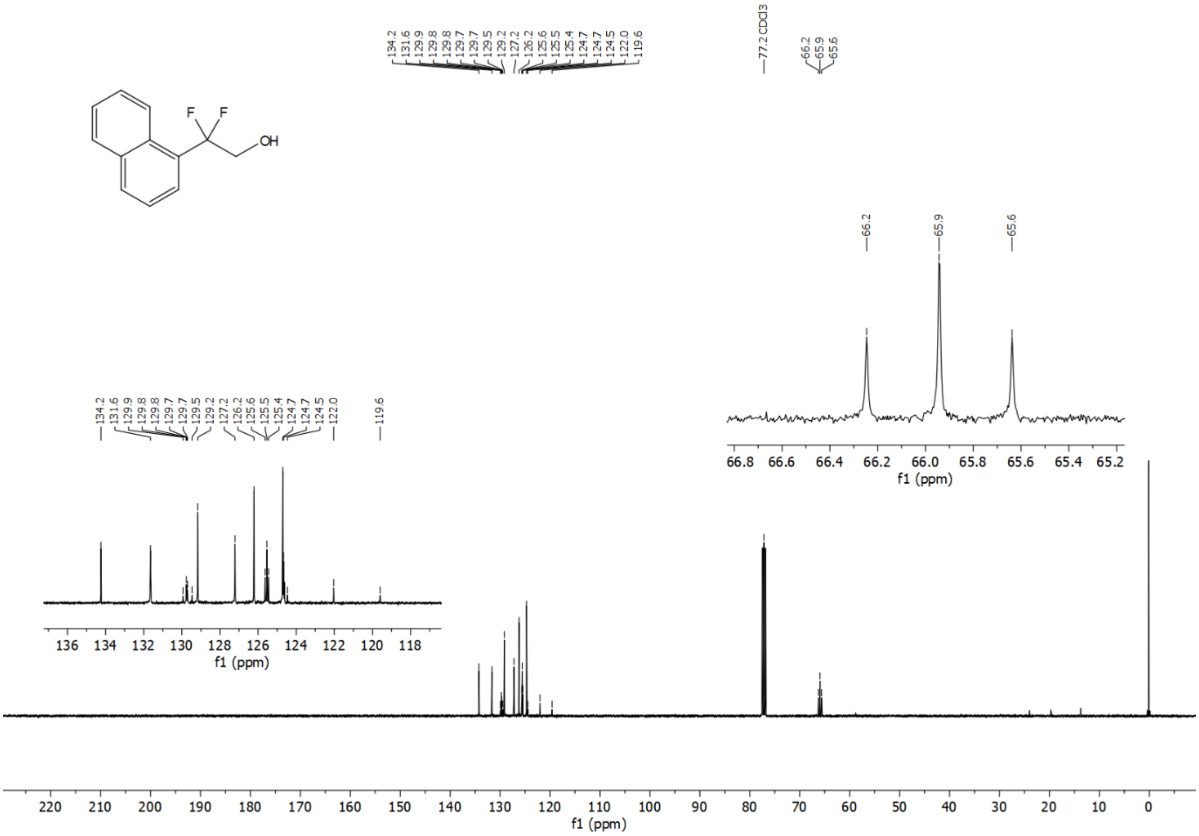

2,2-Difluoro-2-(naphthalen-1-yl)ethan-1-ol (2i) ^{19}F NMR (377 MHz, $CDCl_3$)

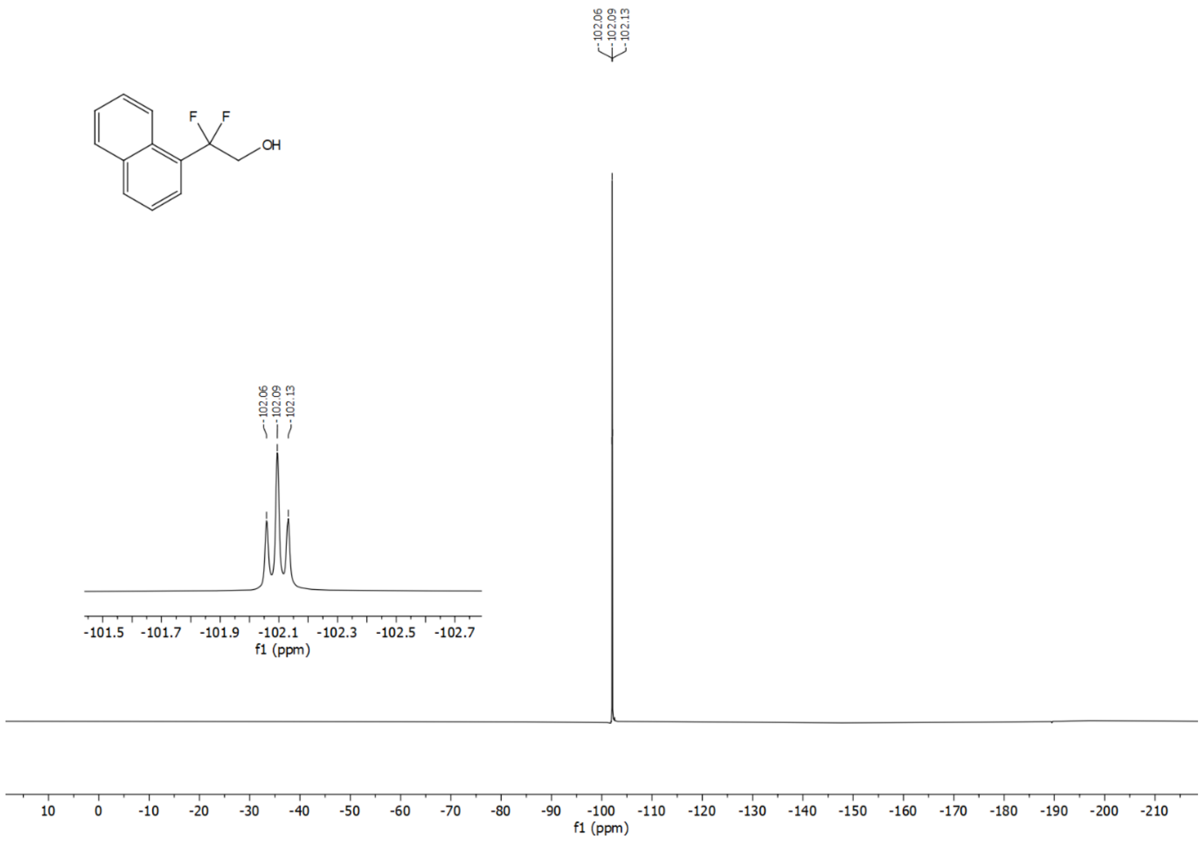

2,2-Difluoro-2-(4-fluorophenyl)ethanol (2j) ^{1}H NMR (400 MHz, $CDCl_3$)

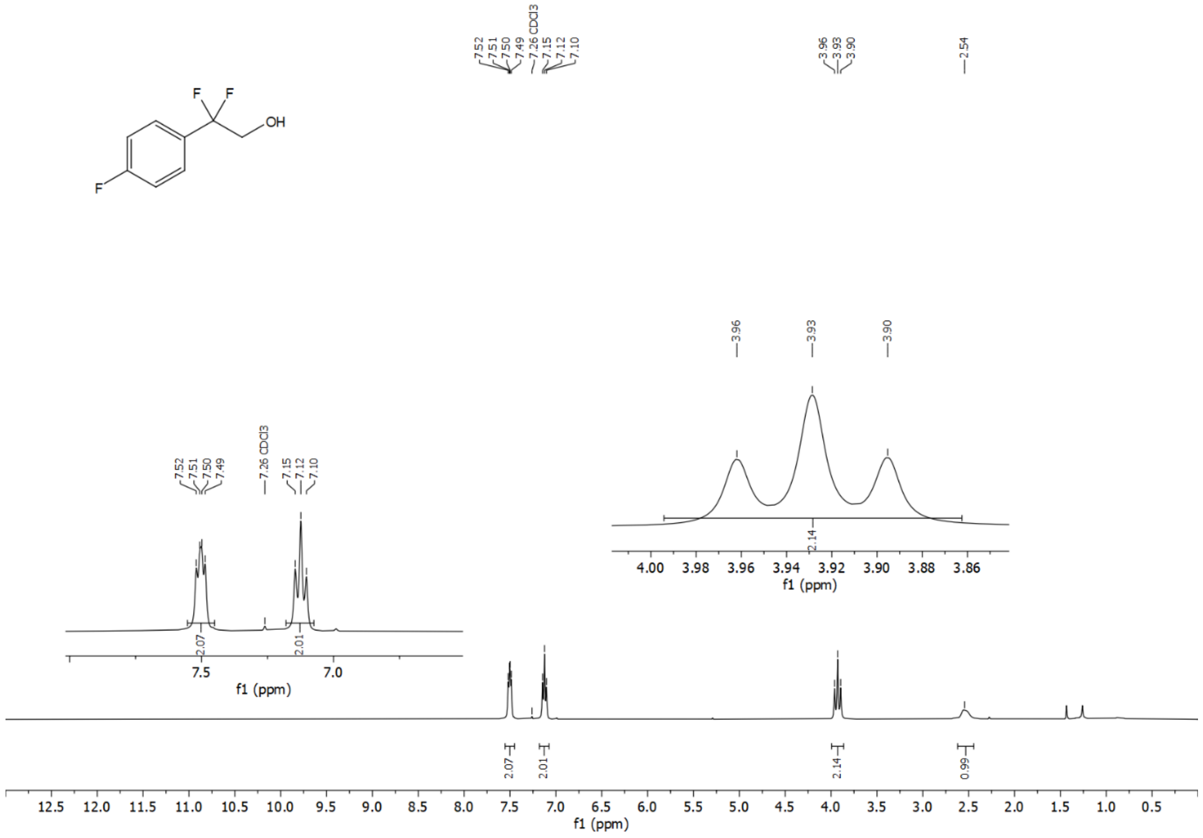

2,2-Difluoro-2-(4-fluorophenyl)ethanol (2j) ^{13}C NMR (100 MHz, $CDCl_3$)

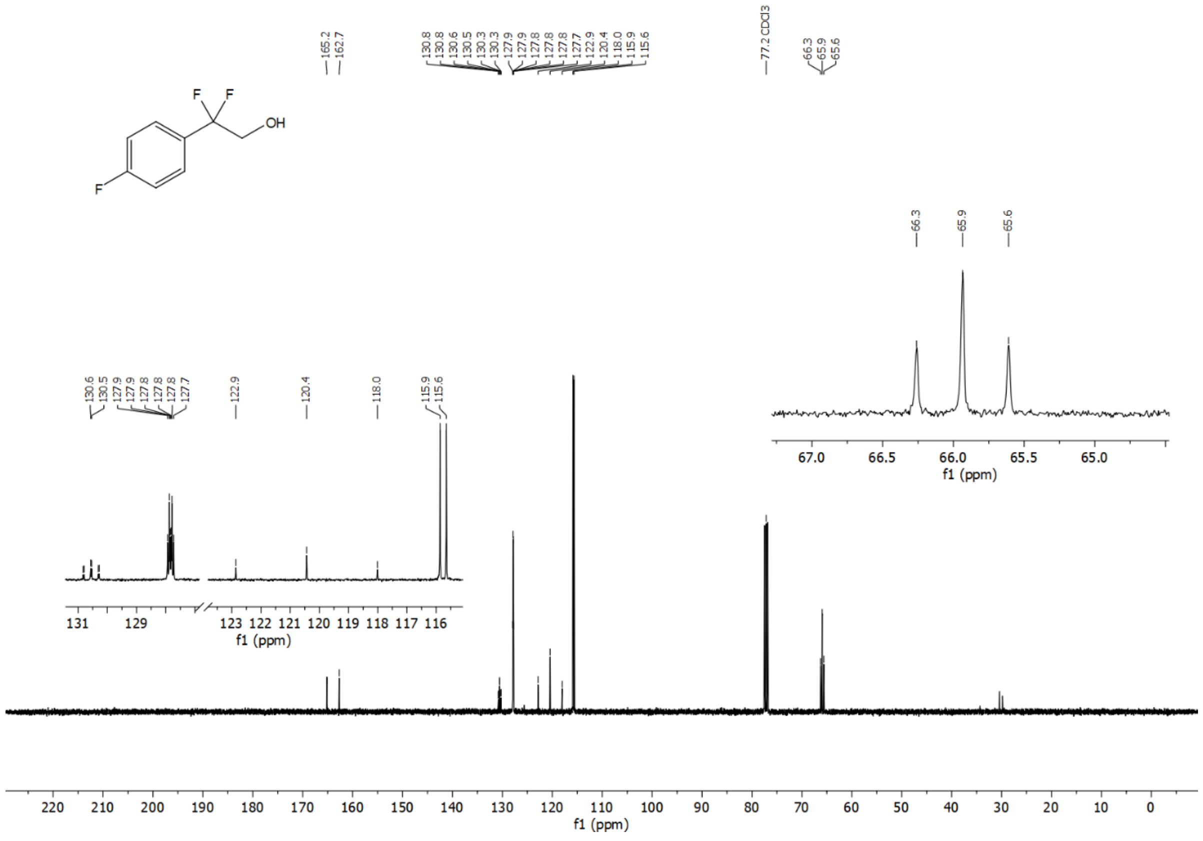

2,2-Difluoro-2-(4-fluorophenyl)ethanol (2j) ^{19}F NMR (377 MHz, $CDCl_3$)

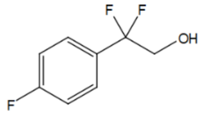

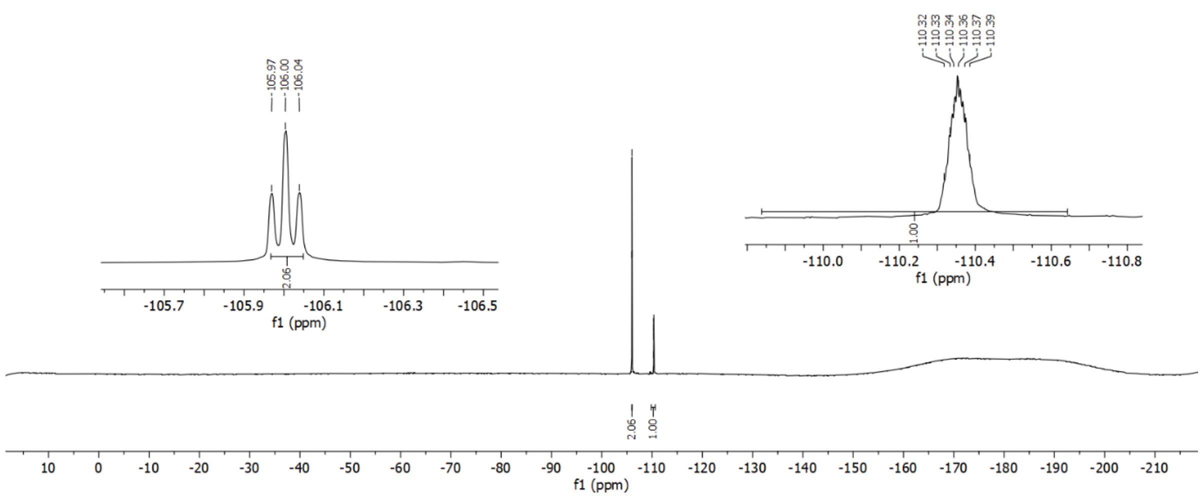

2,2-Difluoro-2-(3-fluorophenyl)ethanol (2k) ^{1}H NMR (400 MHz, $CDCl_3$)

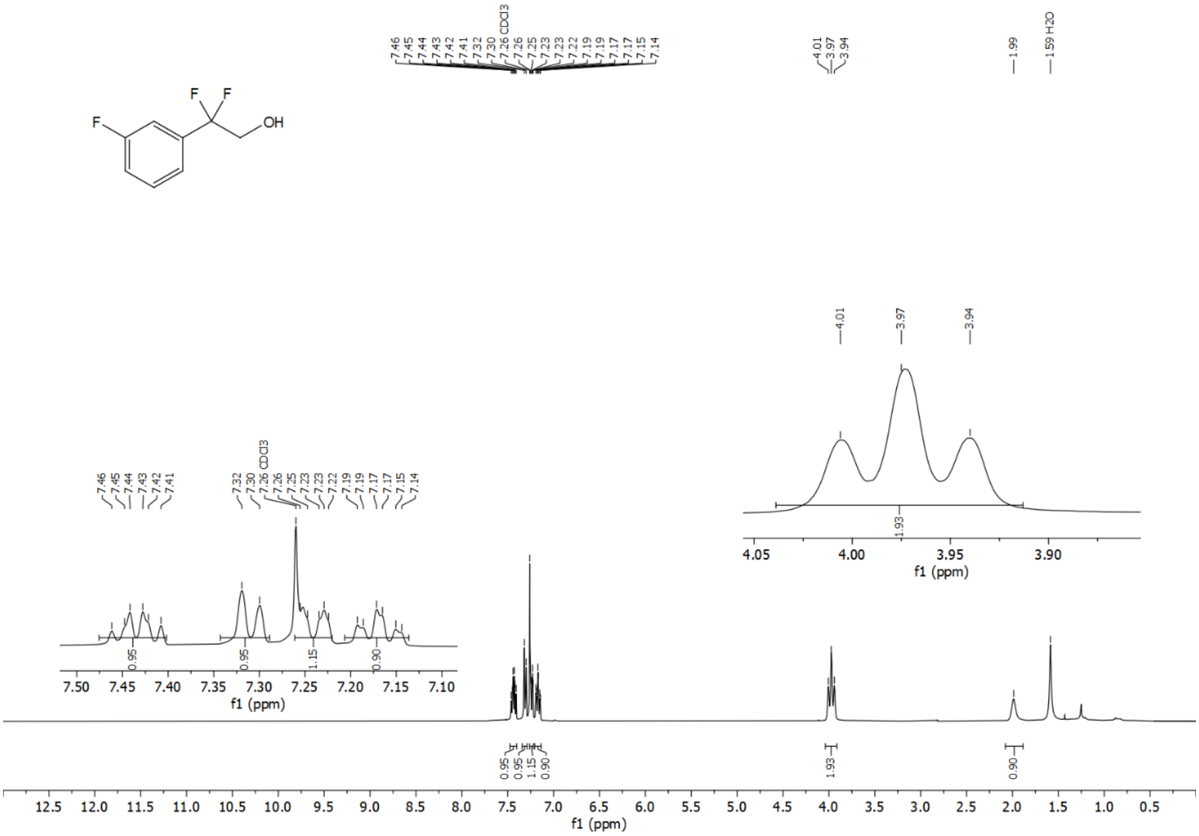

2,2-Difluoro-2-(3-fluorophenyl)ethanol (2k) ^{13}C NMR (100 MHz, $CDCl_3$)

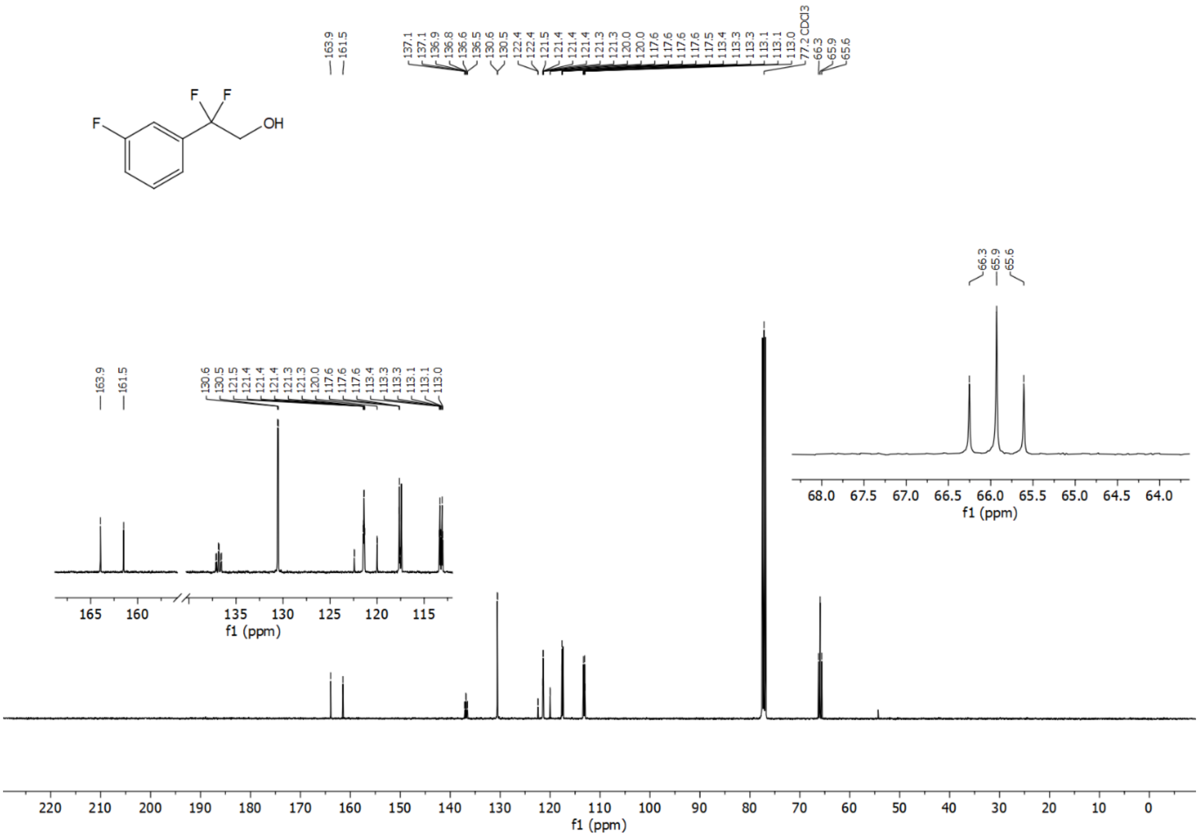

2,2-Difluoro-2-(3-fluorophenyl)ethanol (2k) ^{19}F NMR (377 MHz, $CDCl_3$)

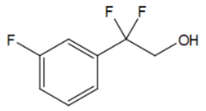

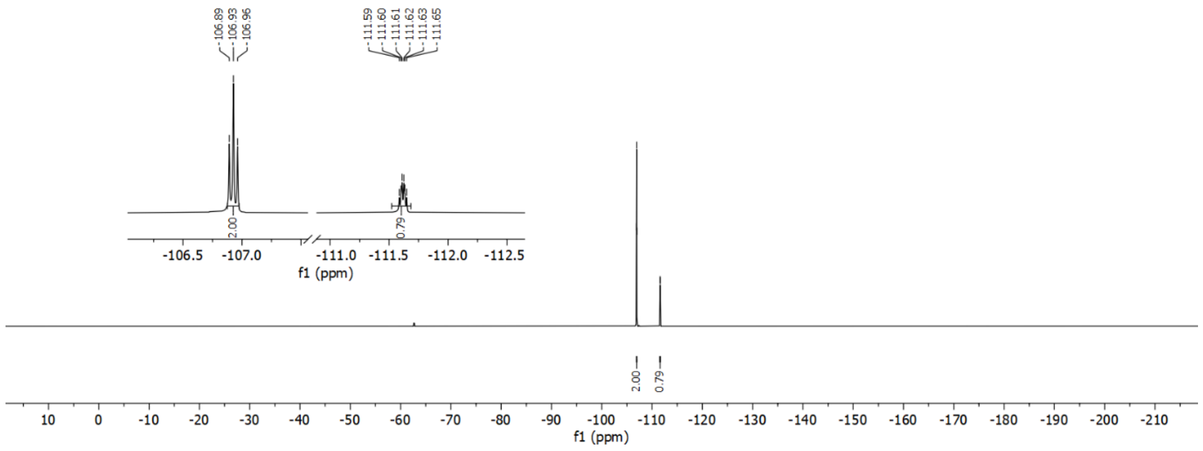

S7 References

1. Li, Y.; Jiang, X.; Zhao, C.; Fu, X.; Xu, X.; Tang, P. *ACS Catal.* **2017**, *7*, 1606–1609.

2. (a) Neese, F. *WIREs Comput. Mol. Sci.* **2011**, *2*, 73–78; (b) Neese, F. *WIREs Comput. Mol. Sci.* **2017**, *8*, 1–6 ; (c) Neese, F.; Wennmohs, F.; Becker, U.; Riplinger, C. *J. Chem. Phys.* **2020**, *152*, 224108; (d) Neese, F. *WIREs Comput. Mol. Sci.* **2022**, *12*, e1606; (e) Neese, F. *WIREs Comput. Mol. Sci.* **2025**, *15*, e70019.

3. Grimme, S.; Hansen, A.; Ehlert, S.; Mewes, J.-M. *J. Chem. Phys.* **2021**, *154*, 064103.

4. (a) Chai, J. D.; Head-Gordon, M. *J. Chem. Phys.* **2008**, *128*, 084106; (b) Lin, Y. S.; Li, G. D.; Mao, S. P.; Chai, J. D. *J. Chem. Theory Comput.* **2013**, *9*, 263–272; (c) Najibi, A.; Goerigk, L. *J. Comput. Chem.* **2020**, *41*, 2562–2572.

5. Weigend, F.; Ahlrichs, R. *Phys. Chem. Chem. Phys.* **2005**, *7*, 3297–3305.

6. van Wüllen, C. *J. Chem. Phys.* **1998**, *109*, 392–399.

7. (a) Neese, F., *J. Comp. Chem.* **2003**, *24*, 1740–1747; (b) Bykov, D.; Petrenko, T.; Izsák, R.; Kossmann, S.; Becker, U.; Valeev, E.; Neese, F. *Molec. Phys.* **2015**, *113*, 1961–1977.

8. Weigend, F. *Phys. Chem. Chem. Phys.* **2006**, *8*, 1057–1065.

9. Marenich, A. V.; Cramer, C. J.; Truhlar, D. G. *J. Phys. Chem. B* **2009**, *113*, 6378–6396.

10. Ásgeirsson, V.; Riplinger, C.; Jónsson, H.*J. Chem. Theory Comput.* **2021**, *17*, 4929–4945.

11. Gherman, B. F.; Heppner, D. E.; Tolman, W. B.; Cramer, C. J. *J. Biol. Inorg. Chem.* **2006**, *11*, 197–205.

12. (a) Haynes, W. M. *CRC Handbook of Chemistry and Physics*, 97th edition, CRC Press, Taylor & Francis Group: Boca Raton, London, New York **2017**; (b) Chirico, R. D.; Frenkel, M.; Diky, V. V.; Marsh, K. N.; Wilhoit, R. C. *J. Chem. Eng. Data* **2003**, *48*, 1344–1359.

13. Schlosser, M.; Brügger, N.; Schmidt, W.; Amrhein, N. *Tetrahedron* **2004**, *60*, 7731–7742.

14. United States patent ”Preparation of pyrrolopyrimidine derivatives as NR2B NMDA receptor antagonists”, US20160075713 A1 2016-03-17

15. United States Patent ” Derivatives of 4-(2-amino-1-hydroxyethyl)phenol as agonists of the β2 adrenergic receptor”, US7964615 B2 2011-06-21

16. Douglas, J. J.; Sevrin, M. J.; Cole, K. P.; Stephenson, C. R. J. *Org. Process Res. Dev.* **2016**, *20*, 1148–1155.

17. Mueller, R.; Rachwal, S.; Lee, S.; Zhong, S.; Li, Y.-X.; Haroldsen, P.; Herbst, T.; Tanimura, S.; Varney, M.; Johnson, S.; Rogers, G.; Street, L. J. *Bioorg. Med. Chem. Lett.* **2011**, *21*, 6170–6175.
